# A genome resource for green millet *Setaria viridis* enables discovery of agronomically valuable loci

**DOI:** 10.1038/s41587-020-0681-2

**Published:** 2020-10-05

**Authors:** Sujan Mamidi, Adam Healey, Pu Huang, Jane Grimwood, Jerry Jenkins, Kerrie Barry, Avinash Sreedasyam, Shengqiang Shu, John T. Lovell, Maximilian Feldman, Jinxia Wu, Yunqing Yu, Cindy Chen, Jenifer Johnson, Hitoshi Sakakibara, Takatoshi Kiba, Tetsuya Sakurai, Rachel Tavares, Dmitri A. Nusinow, Ivan Baxter, Jeremy Schmutz, Thomas P. Brutnell, Elizabeth A. Kellogg

**Affiliations:** 1 HudsonAlpha Institute for Biotechnology, Huntsville, AL USA; 2 Donald Danforth Plant Science Center, St. Louis, MO USA; 3 Department of Energy Joint Genome Institute, Lawrence Berkeley National Laboratory, Berkeley, CA USA; 4 Biotechnology Research Institute, Chinese Academy of Agricultural Sciences, Beijing, China; 5 RIKEN Center for Sustainable Resource Science, Tsurumi, Yokohama, Japan; 6 Present Address: BASF Corporation, Durham, NC USA; 7 Present Address: USDA-ARS Temperate Tree Fruit and Vegetable Research Unit, Prosser, WA USA; 8 Present Address: Graduate School of Bioagricultural Sciences, Nagoya University, Nagoya, Japan; 9 Present Address: Multidisciplinary Science Cluster, Kochi University, Nankoku, Kochi Japan; 10 Present Address: Biology Department, University of Massachusetts, Amherst, MA USA

**Keywords:** Plant biotechnology, Plant breeding, Plant domestication, Natural variation in plants, Plant genetics

## Abstract

Wild and weedy relatives of domesticated crops harbor genetic variants that can advance agricultural biotechnology. Here we provide a genome resource for the wild plant green millet (*Setaria viridis*), a model species for studies of C_4_ grasses, and use the resource to probe domestication genes in the close crop relative foxtail millet (*Setaria italica*). We produced a platinum-quality genome assembly of *S. viridis* and de novo assemblies for 598 wild accessions and exploited these assemblies to identify loci underlying three traits: response to climate, a ‘loss of shattering’ trait that permits mechanical harvest and leaf angle, a predictor of yield in many grass crops. With CRISPR–Cas9 genome editing, we validated *Less Shattering1* (*SvLes1*) as a gene whose product controls seed shattering. In *S. italica*, this gene was rendered nonfunctional by a retrotransposon insertion in the domesticated loss-of-shattering allele *SiLes1-TE* (transposable element). This resource will enhance the utility of *S. viridis* for dissection of complex traits and biotechnological improvement of panicoid crops.

## Abstract (web summary)

Sequencing wild relatives of millet identifies genes that regulate yield and harvesting traits.

## Main

**Fig. 1:**
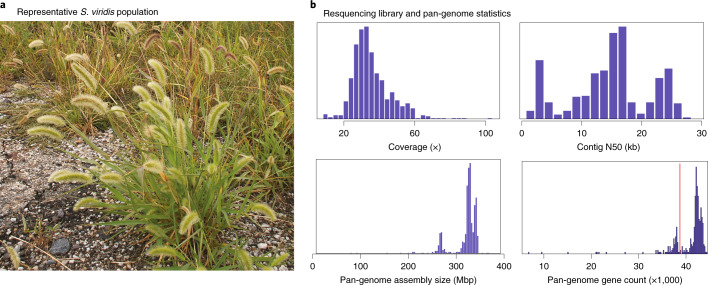
**a**, *S. viridis* in its common, highly disturbed habitat next to a road. **b**, Diversity panel resequencing statistics: average library coverage, contig N50 (Kb), assembly size and number of genes considered present per library. The red vertical line in the lower right panel represents the number of genes necessary for a library to be included for PAV analysis (*n* = 39,000).

Warm-season C_4_ grasses (subfamily Panicoideae), such as maize, sorghum and most species of millet, are mainstays of industrial and small-holder agriculture and include most major biofuel feedstocks. C_4_ photosynthesis is most productive under the hot, dry conditions that are predicted to become more prevalent with climate change^1^. Although contemporary breeding of Panicoideae crops aims to optimize the balance between stress tolerance and yield, de novo domestication of wild Panicoideae species might provide an alternative path to develop new bioproducts, fuels and food sources. However, the plants in the Panicoideae are known for long lifespans and large or complex genomes, creating the need for a practical experimental model that can be used for rapid discovery of gene structure and function and biotechnological improvement of related crops. *S. viridis* (green foxtail) has emerged as such a model^2^. Plants are small (Fig. 1a), diploid, have a short life cycle (seed to seed in 8–10 weeks), a small genome (ca. 500 Mb) and are self-compatible, with a single inflorescence that often produces hundreds of seeds. Transformation is efficient and amenable to CRISPR–Cas9-mediated mutagenesis. These features have enabled the use of *S. viridis* for studies of photosynthetic mechanisms^3^, drought tolerance^4^, cell wall composition^5^, floral and inflorescence development^6^, leaf anatomy^7^, secondary metabolism^8^, plant microbiomes^9^, aluminum tolerance^10^, defense responses^11^ and even inspiration for engineering applications^12^ (Supplementary Fig. 1).

As in most wild species, seeds of *S. viridis* fall off the plant at maturity, a process known as shattering^13^. Although essential for dispersal in natural ecosystems, shattering is undesirable in cultivation, and humans have selected for non-shattering mutants since the dawn of agriculture^14^. Such domesticates include *S. italica* (foxtail millet), the domesticated form of *S. viridis*, which is grown as a crop in Asia^15^. Improvement of some current crops (for example, African fonio and North American wildrice) is hindered because shattering causes high losses at harvest^16^. Thus, identification of proteins controlling shattering have a direct agronomic benefit.

Human selection on wild grasses could have been effective only if there was standing variation in genetic loci that affect shattering. However, to our knowledge, no previous study has succeeded in cloning such loci via association studies of natural diversity. Most previous efforts have, instead, relied on crosses between domesticated plants and their wild progenitors, and genes and quantitative trait loci (QTLs) identified in such studies are, for the most part, not conserved among species^13,17^. We infer that many shattering-related loci remain to be identified.

In this study, we generated genome assemblies for a diverse set of samples (*n* = 598) for *S. viridis*, collected across the United States, analyzed the underlying population structure using single-nucleotide polymorphisms (SNPs) and presence–absence variation (PAV) of individual genes and tested for signatures of selection. We conducted genome-wide association studies (GWASs) and identified previously unknown QTLs for response to the abiotic environment, for a shattering-related locus, *Less shattering1* (*Les1*), and for leaf angle. We validated the function of *Les1* with genome editing. The orthologous gene in *S. italica* is disrupted by a transposon, indicating that the locus contributed to domestication. Our data show that genomics and biotechnological resources in *S. viridis* can be used to accelerate the mechanistic understanding of genetic processes and, thereby, contribute to enhanced and stabilized yield.

## Results

### A complete genome and genomic model for the Panicoideae

We present comprehensive resources for the *Setaria* community. We generated an assembly for the *S. viridis* reference line ‘A10.1’, using a combination of long-read PacBio and Illumina sequencing technologies. The final version 2.0 release is a complete telomere-to-telomere chromosomal assembly, containing 395.1 Mb of sequence in 75 contigs with a contig N50 of 11.2 Mb and a total of 99.95% of assembled bases in nine chromosomes. This is a major improvement over previous *S*. *viridis* genome releases, which had a contig N50 of 1.6 Mb and ca. 94% of reads in contigs. The associated gene annotation is equally complete (BUSCO score^18^ on Embryophyta for the gene set v2.1 is 99%), describing 38,334 gene models and 14,125 alternative transcripts.

To probe the genetic architecture of complex traits, we conducted deep resequencing (mean of 56 million high-quality paired-end (PE) reads, 42.6× coverage) of 598 *S. viridis* diversity samples using Illumina 2 × 150 PE libraries (Fig. 1b; metadata and sequence accession numbers in Supplementary Table 1). Each library was subsequently assembled into a pan-genome database. Although less contiguous than the long read-based A10.1 genome (mean *n* contigs: 75,001; contig N50: 16.2 Kb), the total number of assembled bases was similar (mean assembled bases = 322.5 million) (Fig. 1b).

### Multiple introductions from Eurasia underlie distinct North American gene pools

**Fig. 2:**
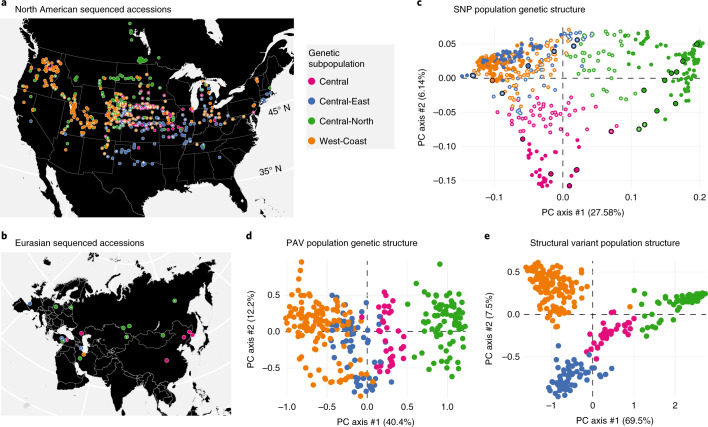
PAV and SNP diversity of subpopulations. **a**, Geographic distribution and population assignment of North American accessions based on SNP data. **b**, Geographic distribution and population assignment of Eurasian accessions based on SNP data. **c**, PCA of SNP data, showing placement of North American samples among Eurasian ones. **d**, PCA of PAV variants, excluding admixed individuals. **e**, PCA of SVs, excluding admixed individuals. *, accession is admixed; black rim on circle, accession is in its native range.

The history of *S. viridis* in North America is unknown, although previous phylogenetic studies place it within a clade of Asian genotypes^19^, indicating that Eurasia is the native range, whereas North America represents a recent and likely human-associated range expansion. To understand the relationship of the North American samples to samples from Eurasia, we called 8.58 million SNPs among our 598 Illumina libraries (Supplementary Table 1). We then extracted polymorphisms similar to the genotype-by-sequencing (GBS) data from previously published Illumina sequence data for 89 non-US samples collected in China, Canada, Europe and the Middle East^20^ and tested for population structure using fastStructure^21^. Overall, we found four distinct subpopulations, all of which are found in both North America and Eurasia (Fig. 2a,b). This pattern is expected if *S. viridis* diversified throughout Eurasia, was introduced to North America from several different sources and then dispersed widely.

To define the evolutionary and biogeographical history of North American *S. viridis*, we conducted an identical population structure analysis with all SNPs in our resequencing panel. Of the 8.58 million SNPs (average, one SNP for every 21.6 bp), 430,000 mapped to exons (primary transcripts), 182,000 SNP were missense, 5,000 were nonsense and 243,000 were silent. As with the GBS data, this analysis identified four main subpopulations (Central, Central-East, Central-North and West-Coast; Supplementary Fig. 2). Not all individuals clustered uniquely into a single subpopulation; 382 genotypes were ‘pure’ and assigned to a single subpopulation, whereas 216 individuals were ‘admixed’ (qi < 0.7). These subpopulations trace their origins to distinct Eurasian gene pools: the ‘Central-East’ subpopulation was closely related to samples from a genetic subpopulation that spanned the northern Middle East region; the closest relatives of the US ‘Central-North’ subpopulation were found from northern Europe through Siberia; the Eurasian members of the ‘West-Coast’ subpopulation were restricted to northern Iran and Afghanistan; and samples from China exclusively group with the ‘Central’ US subpopulation (Fig. 2b,c). The ‘Central’ US subpopulation, whose Eurasian relatives inhabit a range from China to Turkey, had the highest SNP diversity, the highest number of private alleles and the lowest mean linkage disequilibrium (LD) of all the populations (Supplementary Table 2). Such elevated diversity could be driven by larger or multiple founding populations.

### Pan-genome gene PAV mirrors SNP diversity

Intraspecies variation contributes to an organism’s ability to adapt to new environments and respond to biotic and abiotic pressures. Although SNPs serve as excellent proxies of total genetic diversity, adaptation in nature and crop improvement might rely on larger-scale variation, such as gene presence–absence and structural variants (SVs)^22^. Identifying gene gains and losses within a species and their functional annotation will allow for a deeper understanding of adaptation and evolution of the species. To capture the set of all genes in all accessions of *S. viridis* (that is, the pan-genome), we searched for genes that were missing or not annotated in the version 2.1 gene set reference. We identified proteins in *S. italica* (v2.2), *Zea mays* (v.PH207) and *Sorghum bicolor* (v3.1) that were not shared with (that is, not orthologous to) those in *S. viridis* (v2.1) and determined which were present in at least one member of the non-admixed diversity panel (382 accessions). This set of proteins was then added to the set from *S. viridis* (v2.1) to identify a pan-genome of 51,323 genes. Within this pan-genome, a core set of 39,950 genes occurred in 98% of all individuals. Of these core genes, 32,732 were annotated in *S. viridis* (v2.1), with 3,224, 3,412 and 582 more identified based on similarity to *S. italica*, *Z. mays* and *S. bicolor* gene models, respectively. Discriminant function analysis of principal components (DAPC)^23^, a multivariate method for identifying clusters of genes, identified an additional 5,385 genes in 56% of all individuals (the ‘shell’ set) and found the remaining 5,987 genes in 12% of all individuals (the ‘cloud’).

Consistent with other studies of pan-genome population genetics^24^, the SNP- and PAV-based estimates of subpopulation structure are similar. Analysis of the ‘shell’ gene PAV data revealed four subpopulations (*n* = 130, 78, 59 and 35, respectively; Fig. 2d). Of the 203 non-admixed genotypes (that also had sufficient PAV data), 190 (94%) were assigned to the same genetic subpopulation as in the SNP analysis. The ‘Central-North’ subpopulation is clearly distinct in both PAV and SNP data (Fig. 2c), and, when all SVs are considered, the four subpopulations are distinct (Fig. 2e). Among subpopulations, 4,062 genes are significantly over- or under-represented (*P* < 0.05; χ^2^ test; degrees of freedom = 3; critical value = 7.81 with Benjamini–Hochberg correction), with 45 private genes (minor allele frequency (MAF) > 0.1) specific to particular subpopulations (Supplementary Table 3). KEGG and Gene Ontology (GO) enrichment analyses for over-represented genes in each population found pathways relating to biosynthesis of secondary compounds and genes involved in defense response to pathogens or herbivores (Supplementary Fig. 3).

### Selection and correlation with climate in the new range of *S. viridis*

Combined, the PAV and SNP data clearly demonstrate massive genetic diversity and distinct gene pools to target molecular dissection of agriculturally important traits, including response to the environment. Although we have shown that genetic variation in North American *S. viridis* reflects multiple introductions, we have also observed pervasive genetic and biogeographic admixture, suggesting that the North American climate might be filtering and selecting particular combinations of genes and genotypes. Such selection, if present, would be aided by the rapid life cycle and weedy habit of the species. To test for environmental associations, we retrieved the 19 Worldclim^25^ environmental variables for each accession. To overcome collinearity among the climate variables (Supplementary Fig. 4), we used principal components analysis (PCA) to extract the first three principal components (PCs) (36.13%, 30.07% and 16.36% variance explained; factor loadings in Supplementary Table 4), which served as response variables for three GWAS analyses. To control for population structure, we also supplied a set of three SNP-derived PC axes that explain 15.34% of the genetic variation and a kinship matrix to control for relatedness.

Although the first bioclimatic PC axis was not significantly associated with any SNP markers, PC2 had one association (Chr01: 37,770,697) (Supplementary Fig. 5), and PC3 identified several peaks with a total of 140 significant markers (Bonferroni-corrected *P* = 5.82 × 10^−9^). PC3 is loaded by climatic variables relating to extremes of precipitation and temperature. Of the 140 PC3 hits, 66 fall within 16 genes (Supplementary Table 5). Genes within 100 Kbp of the significant markers had products associated with organellar genome maintenance (GO:0033259, GO:0000002 and GO:0006850), protein breakdown (GO:0045732), gibberellic acid homeostasis (GO:0010336) and fucose metabolism (GO:0006004 and GO:0042353) (Supplementary Table 6).

Despite the relatively small number of clear associations with parameters of the abiotic environment, both Tajima’s D and integrated haplotype score (iHS) found multiple genes under selection in the four subpopulations (Supplementary Fig. 6 and Supplementary Table 7). The two tests have different underlying assumptions and methods; genes that appear as outliers in both tests and annotations that arise repeatedly are excellent candidates for further investigation. For example, in most subpopulations, we found selected genes consistently enriched in flavonoid (and other derivatives of phenylalanine) metabolism GO terms and KEGG pathways, processes that often underlie responses to herbivores and pathogens and could be involved in local adaptation. However, selection on other processes and pathways appears to be specific to only one subpopulation. For example, genes whose products are involved in pH reduction in the ‘Central-North’ subpopulation are identified by both tests, although interpretation of this result would require investigation of individual sets of genes and their tissue localization. Together, the bioclim GWAS and tests for selection show that *S. viridis* can provide testable hypotheses of gene function and phenotypic output.

### *SvLes1* controls seed shattering in *S. viridis*

**Fig. 3:**
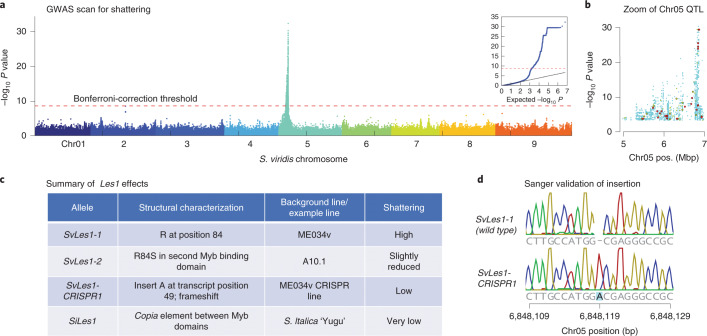
GWAS and cloning of *Les1*. **a**, Manhattan plot of GWAS results, showing a single QTL (peak −log_10_ *P* > 30) for seed shattering on chromosome 5. The red line indicates experiment-wise *P* = 0.01 level after Bonferroni correction. The GWAS used a univariate mixed linear model from GEMMA^40^, with centered kinship matrix. Wald test *P* value was used for assessing significant peaks, but other *P* value estimates give similar results. **b**, Zoom-in on the chromosome 5 peak. Larger dots represent missense SNPs identified by snpEff. Different colors of missense SNPs indicate PROVEAN score range (blue for ≥2.5, green for ≤2.5 and ≥4.1, red for ≤4.1; −2.5 and −4.1 represent 80% and 90% specificity). Lower scores indicate higher likelihood of deleterious effects of the mutation. **c**, Table of *Les1* alleles in *S. viridis* (*Sv*) and *S. itali****c****a* (*Si*), with structural characteristics, background line and shattering phenotype. **d**, Sanger sequence validating the position of the adenine insertion (frameshift) in *SvLes1-CRISPR1*.

We deployed our high-quality genome, pan-genome and population genetic analyses to study the agronomically important phenotype of reduced shattering. We tested a subset of lines for seed shattering using a simple shattering index, in which mature panicles were scored for shattering on a scale of 1 (low) to 7 (high) (Supplementary Table 8). A GWAS identified a single strong QTL (peak −log_10_ *P* > 30) for seed shattering on chromosome 5 (Fig. 3a), a region of approximately 2 Mb above the experiment-wise *P* = 0.01 Bonferroni correction threshold. In this region, 119 mutations, primarily missense, alter protein sequences relative to the reference; we used PROVEAN^26^ to predict deleterious mutations that are more likely to alter the biological function of protein products. Combining this prediction and the association score of SNPs (Fig. 3b), we prioritized a single G-T polymorphism (Chr_05:6849363) in a gene encoding a MYB transcription factor, *SvLes1* (Sevir.5G085400, similar to Sobic.003B087600 from *S. bicolor* and ZM00001d040019_T001 from *Z. mays*), with two MYB DNA-binding domains. The mutation leads to an R84S substitution in the second MYB domain of the protein SvLES1 (Fig. 3c). We named these two alleles *SvLes1-1* and *SvLes1-2*, associated with high seed shattering and reduced shattering, respectively. The *SvLes1-2* allele appears in 24% of the 215 accessions of the GWAS panel and clearly associates with reduced shattering scores (*P* = 6.53 × 10^−33^, two-tailed test; Supplementary Table 8). The reference line A10.1 is among these reduced-shattering lines.

To validate *SvLes1* as the causal gene, we used CRISPR–Cas9 to create additional alleles. We disrupted the wild-type, high-shattering allele *SvLes1-1* in the accession ME034v (corresponding to accession TB0147) to create several nonfunctional alleles. Sequence analysis of *SvLes1-CRISPR1* revealed an adenine insertion at position 149 of the transcript (Fig. 3d), leading to a frameshift mutation predicted to completely abolish gene function, thereby creating non-shattering plants. After segregating out the transgenes encoding Cas9 and guide RNAs, homozygotes of *SvLes1-CRISPR1* were phenotypically examined in the T3 generation.

**Fig. 4:**
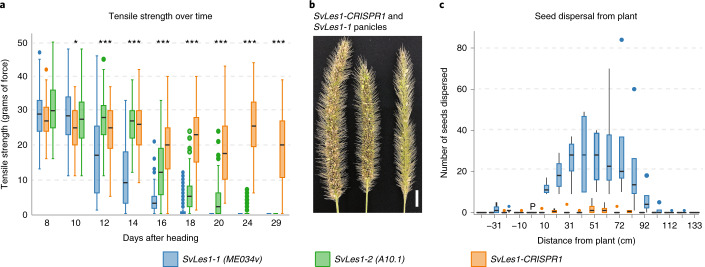
Phenotypic characterization of *SvLes1-CRISPR1* mutants and naturally occurring alleles. **a**, Force required to break the AZ (tensile strength in grams of force) in *SvLes1-1*, *SvLes1-2* and *SvLes1-CRISPR1* lines, measured every 2 d starting at 8 d after heading. For each genotype, *n* = 3 plants (biological replicates) with two inflorescences per plant (technical replicates) and 20 spikelets per inflorescence. Data are presented as median with 25th and 75th percentiles (boxes); whiskers reach values up to 1.5× the interquartile range above and below the hinges; filled circles are outliers. Significance values computed by analysis of variance for each date. *P = 0.0017; ***P < 2.2 × 10^−^^16^. **b**, Image of *S. viridis* high-shattering (*SvLes1-1* in ME034; left two panicles) and *SvLes1-CRISPR1* mutant (right) panicles 7 weeks after heading. Scale bar, 1 cm. **c**, Seed dispersal distances in a wind tunnel, measured from four independent high-shattering plants (*SvLes1-1* in ME034) and eight *SvLes1-CRISPR1* plants at week 6. Data are presented as means ± 1 s.d. (boxes); lines are ± 2 s.d.; filled circles are outliers.

To quantify seed shattering, we measured tensile strength of the abscission zone (AZ)^27,28^. We compared *SvLes1-CRISPR1* and *SvLes1-1* (both in an ME034v background) with *SvLes1-2* (in the A10.1, reduced shattering background). *SvLes1-1* had the lowest tensile strength (high shattering); *SvLes1-2* tensile strength was slightly higher (less shattering); and *SvLes1-CRISPR1* had high tensile strength (reduced seed shattering) (Fig. 4a,b). These results were confirmed with wind tunnel experiments measuring the number of seeds released from the inflorescence and the distance they traveled (Fig. 4c). Few *SvLes1-CRISPR1* seeds were released from the plant, whereas dozens of seeds were released from the *SvLes1-1* plants at 6 weeks after heading. Seeds of the *SvLes1-CRISPR1* allele weighed significantly less than in *SvLes1-1*, but germination percentage was unaffected (Supplementary Fig. 7). T3 offspring of T2 heterozygous plants segregated 3:1 shattering to non-shattering as expected for an induced mutation in a single gene, implying that the lines are isogenic except for the mutant allele.

SvLES1 is a transcription factor that has not been implicated in shattering in any other species. Despite studies identifying shattering-related genes in rice (for example, *Sh4* (ref. ^28^), *qSh1* (ref. ^29^) and *Shat1* (ref. ^30^)) and sorghum (for example, *Sh1* (ref. ^31^)), the cellular mechanism of shattering is not known in any cereal, and recent data suggest that each species might be unique^17^. Unlike other grasses, the AZ in *S. viridis* is not histologically distinct and is only subtly different from that in *S. italica*^13,27^. Cells and cell walls in the AZ are not clearly different from their neighboring cells (demonstrated by multiple different cell wall stains plus TEM^17^). Given this histology, we expected that the AZ of non-shattering mutants would look like that of wild-type plants, and, indeed, the anatomy and histology of *SvLes1-CRISPR1* and *SvLes1-1* spikelets are indistinguishable (Supplementary Fig. 8).

### Recent transposable element insertion in *SiLes1* contributed to domestication of foxtail millet

Because *S. italica* is a domesticated, non-shattering derivative of *S. viridis*^15^, we hypothesized that selection of non-shattering lines could have identified rare alleles of *S. viridis* or *S. italica* early in the domestication process. We discovered a ~6.5-kb *copia* transposable element (*copia38*) inserted between the two Myb domains of *SiLes1* in the *S. italica* line Yugu1. We call this the *SiLes1-TE* allele. The disruption of the Myb domain strongly suggests that *SiLes1-TE* is a loss-of-function allele similar to *SvLes1-CRISPR1* and should also produce a low-shattering phenotype, thus potentially contributing to the domestication of foxtail millet. The *copia38* TE was aligned to each of the 598 *S. viridis* resequenced lines to investigate whether any had the TE insertion in *SvLes1*. Only two samples aligned to the *copia38* TE within the CDS sequence of *SvLes1*, but the nucleotide identity and coverage of the alignments were poor (32% and 6%, respectively). In contrast, *Copia38* is nearly fixed among the foxtail millet lines examined (78 out of 79).

Genome-wide, *S. italica* has about 22% of the SNP variation of *S. viridis* (based on sequences generated in ref. ^32^), as expected given its domestication history. In the *SiLes1* region, however, this number is significantly reduced to 4.1–8.2% of the diversity in *S. viridis*, depending on the size of the region compared (10–100 Kbp; Supplementary Table 9) (*P* = 0.0066 based on 100,000 coalescent simulations of the ratio of π *italica*/π *viridis*). These data hint that the low-shattering QTL might co-localize with a selective sweep. Diversity is relatively high within the *S. italica* gene itself, which might mean that selection is on a regulatory region or additional locus under the QTL or that the transposon insertion has rendered *SiLes1* a pseudo-gene. Estimates of LD support this interpretation (Supplementary Table 10). In *S. viridis*, estimates of LD do not vary much across intervals from 10 to 100 Kbp surrounding *SvLes1*. In *S. italica*, on the other hand, LD is nearly complete for the 20-kb region surrounding *SiLes1*. When that region is expanded to 40 Kbp, LD drops to levels approximating that in *S*. *viridis*. Taken together, these data indicate either a selective sweep or purifying selection on a genomic region that includes *SiLes1* and *copia38* in LD.

*SiLes1* has not been identified in other studies of *S. italica*. Two strong QTLs for shattering were identified in recombinant inbred lines derived from an *S. italica* × *S. viridis* cross (accessions B100 and A10.1, respectively)^33^, but neither QTL encompasses *SiLes1*. Given that A10.1 has the low-shattering allele at *SvLes1*, this result is not surprising. Analysis of 916 diverse accessions of *S. italica* identified 36 selective sweeps, but the *SiLes1* region does not co-localize with any of them^32^. In addition, a previous study^27^ found that tensile strength in two elite *S. italica* lines, Yugu1 and B100, is higher than that of the *SvLes1-CRISPR1* homozygotes reported here. Thus, *SiLes1-TE* is one of several loci contributing to the lack of shattering in foxtail millet.

*Copia38* is a long terminal repeat (LTR) retroelement with two 451-bp LTR sequences. The LTRs for *Copia38* are identical across the entire pan-genome, suggesting that the insertion is recent, because mutations start to accumulate in the once-identical LTRs immediately after insertion of a TE. Phylogenetic analysis showed that *Copia38* tightly clusters with a few homologous copies on a long branch, indicating a shared burst event for copies in this cluster (Supplementary Fig. 9). Pairwise distance among the copies suggests that this burst was recent, on average about 45,000 years ago (range, 23,000–81,000 years), assuming a neutral mutation rate of 6.5 × 10^−9^ bp per year^34^. The copies from this burst occur only in Yugu1 but not in A10.1, suggesting a recent expansion just before domestication of *S. italica* that began approximately 8,000 years ago^15^.

### Parallel genetic control of leaf angle in *S. viridis* and maize by *liguleless2* orthologs

**Fig. 5:**
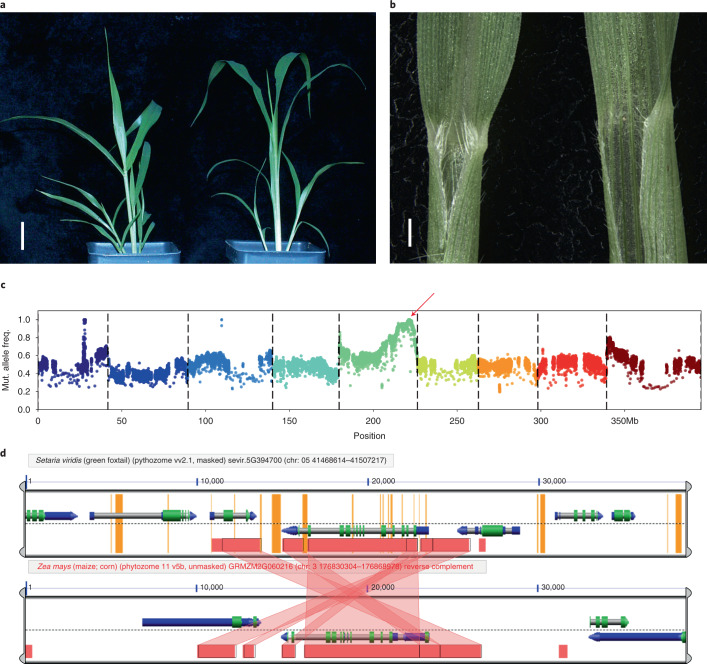
Phenotype and mapping of small leaf angle and loss of ligule. **a**, A10.1 (left) and TB159 (right) showing small leaf phenotype in TB159 relative to A10.1. Scale, 2 cm. **b**, Junction of leaf and blade for A10.1 (left) and TB159 (right) showing well-developed ligule in A10.1 and lack of ligule in TB159. Scale, 5 mm. **c**, BSA mapping result, red arrow indicating QTL. Wald test *P* value was used to assess significance, but other *P* value estimates give similar results. **d**, Synteny analysis around *SvLg2* and maize *lg2* locus. Screenshot from analysis with CoGE (https://genomevolution.org/r/1ei1s).

We discovered a single accession in the panel (TB0159) with reduced auricle and ligule development and markedly upright leaves (small leaf angle) (Fig. 5a,b). As a GWAS is not suitable for mapping traits with low frequency and strong effects, bulked segregant analysis (BSA)^6,20^ was used to identify the associated genomic region. In BSA, plants with and without a particular phenotype are pooled (bulked), and each pool is sequenced. Regions or loci that differ between the two pools are then inferred to contain the mutations underlying the phenotype. TB0159 was crossed to A10.1, and the F_1_ plants showed wild-type leaf angle, showing that small leaf angle is recessive. The wild-type and small leaf angle trait in the F_2_ population segregated at 264:153, which differs significantly from a 3:1 ratio (*P* = 0.000238). This could be explained by partial dominance at a single locus or by several loci controlling the phenotype. We proceeded assuming a single partially dominant causal gene for small leaf angle.

With BSA, we coarsely mapped the reduced leaf angle phenotype to a homozygous region of ~800 kb on chromosome 5 (Fig. 5c) that contained 104 disruptive SNPs and 687 indels (393 single bp). This region includes *SvLiguless2* (*SvLg2*) (Sevir.5G394700), the syntenic ortholog of *liguless2* in maize^35^ (Fig. 5d), which is a transcriptional regulator that controls auricle and ligule development and leaf angle^36^. Because the small leaf angle phenotype is partially dominant and unique to TB0159 in the panel, the causal allele should be homozygous and occur only in that accession. We identified a homozygous G insertion (Chr_5:41489494) in the coding region of *SvLg2* that causes a frameshift, supporting the hypothesis that SvLG2 indeed controls ligule development in *S. viridis* as it does in maize.

## Discussion

With changes in climate, it becomes essential to understand the genetics of domestication of wild species, which has application for plant breeding and crop improvement. In this study, we provided a high-quality genome of *S. viridis*, a model C_4_ plant and a wild progenitor of domesticated foxtail millet (*S. italica*). Multiple sources of evidence support that *S. viridis* has an ancestral range in Eurasia^19^, was introduced to the United States via multiple routes and subsequently dispersed within the United States. The resequenced accessions described here provide a rich source of variants (SNP, PAV and SV) that reflect the population structure of the species in the United States. We found relatively few loci associated with variation in the abiotic environment in environmental GWASs. However, tests for selection found numerous genes under selection, notably those in GO and KEGG categories encompassing metabolism of flavonoids and other derivatives of phenyalanine, which could indicate adaptation to the biotic environment. This result is consistent with a recent study of the *S. viridis* accession ME034v and a subset of the diversity lines studied here, which discovered expansion of gene families involved in specialized metabolism and defense response^37^.

Our PAV data connect published QTL studies with population-level processes. For example, a study of drought response in *S. viridis* found a strong-effect QTL significant for plant size, water loss and water use efficiency across all tested environments^38^. Investigation within the pan-genome found an S-phase kinase associated protein 1 (*Skp1)* homolog (Sevir.2G407800) within 50 kb of the associated SNP that was common in the ‘Central-North’ subpopulation (present in 65% of individuals) whereas significantly under-represented in the ‘Central-East‘ (present in 5% of individuals) (*P* < 4.3 × 10^−15^). SKP1 proteins are part of the E3 ubiquitin ligase complex that leads to protein degradation^39^. Whereas the physiological role of this particular SKP1 homolog is unknown, the fact that it falls within a known QTL interval and is differentially represented in two populations suggests that it might be a candidate for future investigation.

Shattering has been an important trait in agriculture from the dawn of domestication. We identified a gene using association mapping and validated *SvLes1* using CRISPR–Cas9. A frameshift mutation completely abolished gene function, thereby creating non-shattering plants. In *S. italica*, the ortholog has a *copia38* element inserted between two Myb domains, which leads to a loss of function. A plausible scenario is that the *SiLes1-TE* allele was a low-frequency allele that was selected approximately 8,000 years ago during domestication owing to its favored low-shattering phenotype and spread quickly through the early foxtail millet land races. Later, the low-shattering phenotype was further strengthened by additional loci with stronger effects (that is, SvSh1 (refs. ^32,33^)) during recent crop improvement. The engineered allele *SvLes1-CRISPR1* allows us to recreate a low-shattering phenotype from ancestral *S. viridis* alleles, mimicking the initial phase of foxtail millet domestication.

## Methods

### Plant materials

The reference line A10.1 is a descendant of the line used by Wang et al.^41^ in early restriction fragment length polymorphism maps. The original line was found to be heterozygous, and, thus, A10.1 was propagated via single-seed descent by Andrew Doust (Oklahoma State University; personal communication). It is thought to have originated in Canada. The other reference, ME034 (also known as ME034v), was collected by Matt Estep (Appalachian State University) in southern Canada as part of a diversity panel^19^ included among the diversity lines sequenced here; its genome has also been assembled recently^37^. Transformation is more efficient for ME034 than for A10.1^37^, and, thus, the latter is being used widely for functional genetic studies.

The 598 individuals of the diversity panel were collected over a period of several years. About 200 lines were described in previous studies^19,42^, whereas others were new collections added for this project. Individuals were propagated by single-seed descent, although the number of generations varies by accession. *S. viridis* is inbreeding by nature (ca. 1%^42^), so we assume that initial heterozygosity was generally low and then further reduced in propagation.

### Library creation and sequencing

To prepare DNA for sequencing of the reference line, 100 ng of DNA was sheared to 500 bp using the Covaris LE220 (Covaris) and size selected using SPRI beads (Beckman Coulter). The fragments were treated with end-repair, A-tailing and ligation of Illumina compatible adapters (IDT) using the KAPA-Illumina Library Creation Kit (KAPA Biosystems). The prepared library was then quantified using KAPA Biosystems’s next-generation sequencing library quantitative polymerase chain reaction (qPCR) kit and run on a Roche LightCycler 480 real-time PCR instrument. The quantified library was then multiplexed with other libraries, and the pool of libraries was prepared for sequencing on the Illumina HiSeq sequencing platform using a TruSeq PE Cluster Kit (v3) and Illumina’s cBot instrument to generate a clustered flowcell for sequencing. Sequencing of the flowcell was performed on the Illumina HiSeq2000 sequencer using a TruSeq SBS Sequencing Kit (v3) following a 2 × 150 indexed run recipe.

Plate-based DNA library preparation for Illumina sequencing was performed on the PerkinElmer Sciclone NGS robotic liquid handling system using KAPA Biosystems’s library preparation kit. Next, 200 ng of sample DNA was sheared to 600 bp using a Covaris LE220 focused ultrasonicator. Sheared DNA fragments were size selected by double SPRI, and then the selected fragments were end-repaired, A-tailed and ligated with Illumina compatible sequencing adaptors from IDT containing a unique molecular index barcode for each sample library.

The prepared library was quantified using KAPA Biosystems’s next-generation sequencing library qPCR kit and run on a Roche LightCycler 480 real-time PCR instrument. The quantified library was then multiplexed with other libraries, and the pool of libraries was then prepared for sequencing on the Illumina HiSeq sequencing platform using a TruSeq PE Cluster Kit (v3 or v4) and Illumina’s cBot instrument to generate a clustered flowcell for sequencing. Sequencing of the flowcell was performed on the Illumina HiSeq2000 or HiSeq2500 sequencer using HiSeq TruSeq SBS Sequencing Kits (v3 or v4) following a 2 × 150 indexed run recipe.

### Sequencing of the reference genome

We sequenced *S. viridis* A10.1 using a whole-genome shotgun sequencing strategy and standard sequencing protocols. Sequencing reads were collected using Illumina HISeq and PacBio Sequel platforms at the Department of Energy Joint Genome Institute in Walnut Creek, California, and the HudsonAlpha Institute for Biotechnology in Huntsville, Alabama. One 800-bp insert 2 × 250 Illumina fragment library (240×) was sequenced, giving 425,635,116 reads (Supplementary Table 11). Illumina reads were screened for mitochondria, chloroplast and PhiX contamination. Reads composed of greater than 95% simple sequence were removed. Illumina reads less than 75 bp after trimming for adapter and quality (*q* < 20) were removed. For the PacBio sequencing, a total of 36 P5C2 chips (4-h movie time) and 41 P6C4 chips (10-h movie time) were sequenced with a p-read yield of 59.09 Gb, with a total coverage of 118.18× (Supplementary Tables 11 and 12).

### Genome assembly and construction of pseudomolecule chromosomes

An improved version 2.0 assembly was generated by assembling 4,768,857 PacBio reads (118.18× sequence coverage) with the MECAT assembler^43^ and subsequently polished using QUIVER^44^. The 425,635,116 Illumina sequence reads (240× sequence coverage) were used for correcting homozygous SNP/indel errors in the consensus. This produced 110 scaffolds (110 contigs), with a contig N50 of 16.8 Mb and a total genome size of 397.9 Mb (Supplementary Table 13). A set of 36,061 syntenic markers derived from the version 2.2 *S. italica* release was aligned to the MECAT assembly. Misjoins were characterized as a discontinuity in the *italica* linkage group. A total of 15 breaks were identified and made. The *viridis* scaffolds were then oriented, ordered and joined together into nine chromosomes using syntenic markers. A total of 61 joins were made during this process. Each chromosome join is padded with 10,000 Ns. Significant telomeric sequence was identified using the TTTAGGG repeat, and care was taken to make sure that it was properly oriented in the production assembly.

Scaffolds that were not anchored in a chromosome were classified into bins depending on sequence content. Contamination was identified using blastn against the National Center of Biotechnology Information nucleotide collection (NR/NT) and blastx using a set of known microbial proteins. Additional scaffolds were classified as repetitive (>95% masked with 24mers that occur more than four times in the genome) (26 scaffolds, 1.2 Mb), alternative haplotypes (unanchored sequence with >95% identity and >95% coverage within a chromosome) (15 scaffolds, 1.0 Mb), chloroplast (three scaffolds, 164.5 Kb), mitochondria (five scaffolds, 344.9 Kb) and low quality (>50% unpolished bases after polishing, one scaffold, 19.3 Kb). The resulting final statistics are shown in Supplementary Table 14.

Finally, homozygous SNPs and indels were corrected in the release consensus sequence using ~60× of Illumina reads (2 × 250, 800-bp insert) by aligning the reads using bwa mem^45^ and identifying homozygous SNPs and indels with the GATK’s UnifiedGenotyper tool^46^. A total of 96 homozygous SNPs and 4,606 homozygous indels were corrected in the release. The final version 2.0 release contains 395.1 Mb of sequence, consisting of 75 contigs with a contig N50 of 11.2 Mb and a total of 99.95% of assembled bases in chromosomes (Supplementary Table 14).

Completeness of the version 2.0 assembly is very high. To further assess completeness of the euchromatic portion of the version 2.0 assembly, a set of 40,603 annotated genes from the *S. italica* release was used for comparison. The aim of this analysis was to obtain a measure of completeness of the assembly rather than a comprehensive examination of gene space. The transcripts were aligned to the assembly using BLAT, and alignments ≥90% base pair identity and ≥85% coverage were retained. The screened alignments indicate that 39,441 (97.14%) of the *italica* genes aligned to the version 2.1 release. Of the unaligned 1,162 transcripts, 928 (2.28%) indicated a partial alignment, and 234 (0.58%) were not found in the version 2.1 release.

To assess the accuracy of the assembly, a set of 335 contiguous Illumina clones >20 Kb was selected. A range of variants was detected in the comparison of the clones and the assembly. In 239 of the clones, the alignments were of high quality (<0.01% bp error) with an example given in Supplementary Fig. 10a (all dot plots were generated using Gepard^47^). The remaining 96 clones indicate a higher error rate due, mainly, to their placement in more repetitive regions (Supplementary Fig. 10b). The major component of the error in the 96 repetitive clones was copy number variation, which affected 50 of the clones. These 50 clones accounted for more than 97% of all of the errors in the 335-clone set. Excluding the clones with copy number variation, the overall base pair error rate in the 285-clone set is 0.0098% (1,043 discrepant base pairs out of 10,601,785).

### Annotation

Various Illumina RNA sequencing reads were used to construct transcript assemblies using a genome-guided assembler, PERTRAN^48^: 1 billion pairs of geneAtlas, 0.9 billion pairs of LDHH, 0.9 billion pairs of LLHC and 176 million other pairs. Next, 109,119 transcript assemblies were constructed using PASA^49^ from RNA sequencing transcript assemblies above. Gene models of version 1.1 on assembly version 1.0 were lifted over to assembly version 2.0 and improved. The in-house gene model improvement pipeline is as follows:

The genomic sequence of a locus is obtained, including introns, if any, and up to 1-Kbp extensions on both ends unless they run into another gene. For intergenic space less than 2 Kbp, half of the intergenic distance is the extension for two adjacent loci. These locus sequences are mapped to a new genome using BLAT. Duplicate mappings are resolved using the gene model’s neighbors in original genome space. When a locus genomic sequence is mapped to the new genome uniquely and 100%, the gene model is perfectly transferred to the new genome. For the remaining gene models, both their transcript and CDS sequences are mapped with BLAT to the region in the new genome located by locus genomic sequence mapping above. Gene models are made from CDS alignments with quality of 95% identity, 90% coverage and valid splice sites, if any, and are transferred if the resulting peptide is 70% or more similar to the original gene model peptide. Untranslated regions (UTRs), if any, are added using transcript alignments. Remaining gene models are mapped to the new genome using GMAP. Gene models based on GMAP alignments with quality of 95% identity, 70% coverage and valid splice sites, if any, are transferred if, and only if, the resulting gene model peptide is 70% or more similar to the original gene model peptides and in the new genome location not occupied by transferred gene models in earlier steps.

Non-overlapping complete open reading frames (ORFs) from each PASA transcript assembly (TA) were predicted if the ORF had good homology support or was long enough (300 bp if multi exons or 500 bp if single exon). Proteins from *Arabidopsis thaliana*, rice, sorghum, *Brachypodium distachyon*, *S. italica*, grape, soybean and Swiss-Prot eukaryote were used to score TA ORFs using BLASTP. The TA ORFs were then fed into the Program to Assemble Spliced Alignments (PASA) pipeline where EST assemblies were obtained for gene model improvement, including adding UTRs. PASA-improved gene model transcripts were compared to version 1.1 lifted-over models on how well the transcript CDS was supported by ESTs and/or homologous protein and not overlapped with repeats generated with RepeatMasker (http://www.repeatmasker.org/) for more than 20%. If PASA gene models of TA ORFs were better than lifted-over ones, the PASA gene models took over the lifted-over ones. Otherwise, the lifted-over gene model stayed. The final gene model proteins were assigned to protein families using PFAM and PANTHER, and gene models were further filtered for those with 30% or more of proteins assigned to transposable element domains.

Locus model name was assigned by mapping forward the version 1.1 locus model, if possible, using our locus name map pipeline; otherwise, a new name was given using the Joint Genome Institute locus naming convention that was used in version 1.1 locus model naming. Our locus name map pipeline is as follows: a locus is said to be mapped and name mapped forward if 1) the previous version and current version loci overlap uniquely and appear on the same strand; and 2) at least one pair of translated transcripts from the old and new loci are mutual best hits (MBHs) with at least 70% normalized identity in a BLASTP alignment (normalized identity defined as the number of identical residues divided by the longer sequence). For a given pair of previous version and current version transcripts at mapped loci, transcript model names are mapped forward if either 1) an MBH relationship exists between the two proteins with at least 90% normalized identity; or 2) the proteins have at least 90% normalized identity and are not MBHs, but the corresponding transcripts sequences are (also with 90% normalized identity). This latter rule is specifically to handle cases where the previous version and current version models differ mainly by the addition of, or extension of, the UTR to a previous version model. These rules allowed the model names of approximately 92% of version 1.1 gene models to be mapped forward to version 2.1.

### Sequencing and assembly of the diversity panel

After excluding seven lines because of low sequence coverage, 598 diversity samples (metadata, including Sequence Read Archive numbers, in Supplementary Table 1) were used for diversity analysis. The samples were sequenced using Illumina PE sequencing (2 × 151 bp) at the Department of Energy Joint Genome Institute and the HudsonAlpha Institute for Biotechnology using Hiseq 2500 and NovoSeq6000. Individual de novo assemblies for each line were constructed using Meraculous (v2.2.5)^50^ with a k-mer size of 51, selected to maximize the contig N50 in the resultant assemblies and to ensure that alternative haplotypes would have the best chance of being split apart. To construct chromosomes for each library, exons from the *S. viridis* gene set reference (v2.1; number of genes = 38,334; number of exons = 289,357) were aligned to each Meraculous assembly (blastn, word_size = 32), and exon alignments with identity ≥90% and coverage ≥85% were retained. Scaffolds were joined into gene-based scaffolds based on exon alignments; synteny and exon alignments were then used to order and orient the sequences into chromosomes (Supplementary Fig. 11).

### SNP calling

Reads from the diversity samples were mapped to *S. viridis* version 2.1 using bwa-mem^45^. The bam was filtered for duplicates using Picard (http://broadinstitute.github.io/picard) and realigned around indels using GATK^46^. Multi-sample SNP calling was done using SAMtools mpileup and Varscan V2.4.0 (ref. ^51^) with a minimum coverage of 8 and a minimum alternate allele frequency of 4. An allele is confirmed to be homozygous or heterozygous using a binomial test for significance at a *P* value of 0.05. Repeat content of the genome was masked using 24-bp k-mers. k-mers that occur at a high frequency, up to 5%, were masked. SNPs around 25 bp of the mask were removed for further analysis. An SNP was included for further analysis if it had a coverage in 90% of the samples and a MAF > 0.01. Imputation and phasing were done in Beagle V4.0. SNP annotation was performed using snpEff^52^.

### Pan-genome and PAV analysis

To assess PAV of genes across the diversity panel, admixed individuals were removed from the analysis, leaving 382 individuals (Q ≥ 0.7). We expected that some genes present in wild accessions of *S. viridis* (that is, the pan-genome) would be either missing or not annotated in the version 2.1 reference gene set. To capture these, we included not only proteins from *S. viridis* (v2.1 gene set) but also non-orthologous proteins from *S. italica* (v2.2), *Z. mays* (v.PH207) and *S. bicolor* (v3.1) (based on InParanoid comparisons^53^) and aligned these to chromosome integrated assemblies from each of the four subpopulations using BLAT (-noHead -extendThroughN -q=prot -t=dnax).

Genes from *S. viridis* and *S. italica* were considered present if they aligned with more than 85% coverage and identity or at least 90% coverage and identity if the exons were broken up and located on no more than three contigs. *S. bicolor* and *Z. mays* genes were considered present if they aligned with more than 70% identity and 75% coverage (to allow for greater divergence among sequences) or at least 80% identify and coverage if the exons were broken up and located on no more than three contigs. Libraries with fewer than 39,000 genes considered present were excluded as genes were likely lost owing to low coverage/poor assembly, with 302 individuals remaining for analysis. Then, 67,079 genes were aligned to each assembly. After removing genes that aligned poorly or not at all to any assembly, a total of 51,323 genes were retained. The resultant PAV matrix (Supplementary Table 15) was used to determine and cluster genes into their pan-genome designation (core, shell and cloud) using DAPC^23^. Using successive k-means clustering, three distinct clusters (based on the Bayesian information criterion (BIC)) were discovered based on their PAV observance across non-admixed individuals, designated core, shell and cloud. Genomic coordinates for non-orthologous proteins in the pan-genome were determined using the GENESPACE pipeline^48^.

To discover which genes were over- or under-represented within each subpopulation based on their expected observance, a χ^2^ test (with Benjamini–Hochberg correction) was performed for each gene among each of the four subpopulations. Significantly over-represented genes (allowing overlap among subpopulations; *P* < 0.05) within subpopulations were also tested.

To infer syntenic regions when placing non-orthologous genes of interest back on the *S. viridis* genome, we applied the GENESPACE pipeline^48^ to the *S. viridis* genome described herein and five other grasses: *S. italica* (v2.2), *S. bicolor* (v3.1), *Oryza sativa* (‘Kitaake’, v3.1), *Z. mays* (‘Ensemble-18’) and *B.* *distachyon* (v3.1). Genome annotations and assemblies were downloaded from phytozome (https://phytozome.jgi.doe.gov/pz/portal.html).

### SVs

To detect SVs within the pan-genome, pseudo PacBio reads were generated from assemblies of non-admixed individuals. Pseudo-reads (length: 10 kb, depth: 5×) were generated from all contigs greater than 10 kb from each pan-genome assembly. The pseudo-reads were aligned to the *S. viridis* reference genome using nglmr (v0.2.7)^54^ with default settings for PacBio reads. The resulting bam file was sorted using samtools (v1.10) and used for calling SVs with sniffles (v1.0.11). The SV types considered across the pan-genome were: insertions, deletions and inversions. The average number of SVs detected per library was 15,593. A presence–absence matrix for each SV type and was clustered using iterative k-means and BIC to determine goodness of fit and the optimal number of clusters present. Based on the BIC, there were three main clusters within the PAV matrix, the first being likely false positives (mean observance, 2.8%; *n* = 163,199). The second and third clusters were combined (mean observance, 33%; *n* = 33,350) and used to calculate a distance matrix (method-jaccard) for visualizing subpopulation differences on a PCA plot (Fig. 2e).

### Population structure

Population structure for both SNP and PAV data was estimated using fastStructure^21^. SNP markers were randomly subsetted to 50,000 by LD pruning (parameters: --indep-pairwise 50 50 0.5) in plink 1.9 (ref. ^55^), whereas shell genes (as determined by DAPC clustering) were extracted from the pan-genome. In both analyses, a single sample with a maximum membership coefficient (qi) of <0.7 was considered admixed. Only non-admixed samples from the SNP analysis were used for further analysis. For SNP markers, multidimensional scaling, identity by state and LD estimates (parameters: --r2 --ld-window-kb 500 --ld-window-r2 0) were performed in plink 1.9.

### Extraction of GBS markers from diversity panel assemblies

To integrate previously published GBS data^42^ with our new sequencing data, we extracted the relevant GBS markers from the assembled genomes. The PE sequences were demultiplexed with the sabre package (https://github.com/najoshi/sabre). Samples were aligned to the reference using bwa-mem, and SNPs were further called using Varscan 2.4.0 (minimum depth of 3 and variant allele depth of 2). SNPs with more than 20% missing data in the GBS data were removed, and then those remaining were merged with the diversity panel (598 samples with 8.58 million markers). A common set of 55,360 SNPs was obtained.

### LD decay

To determine the extent of LD in the population, first we extracted one SNP every 100 bp using plink (--bp-space 100) and selected a random set of 200,000 markers. LD (r^2^) was calculated using plink (--ld-window 500 --ld-window-kb 2000). The r^2^ value was averaged every 100 bp of distance. A nonlinear model was fit for this data in R, and the extent was determined as when the LD (r^2^) nonlinear curve reached 0.2. Average LD was 100 Kbp. This distance defined the window size for searching for candidate genes in the GWAS analyses.

### Search for *copia* elements

The *copia38* sequence (6.7 kb) was extracted from the genomic sequence of Seita.5G087200 using repbase (https://www.girinst.org/repbase/). Both the *copia38* sequence and the *SvLes1* (Sevir.5G085400) sequence (both genomic sequence and CDS) were aligned to each of the pan-genome assemblies (*n* = 598) using BLAT (-noHead -extendThroughN). From the BLAT results, each *copia38* alignment was checked whether it fell within the bounds of the *SvLes1* locus.

### Environmental correlations

Climate data were obtained from WorldClim^25^ using the Raster package in R for each of the 577 samples that have geographical coordinates. Correlations were calculated and visualized using the corrplot R package. To account for correlations between the 19 bioclimatic variables, we performed PCA using prcomp in R. The top three PCs that contributed most of the variance were used independently as response variables in the association analysis. GEMMA^40^ was used to identify the association of bioclimatic variables and each of the SNPs using only kinship in one model and using both kinship and population structure in another model. For population structure estimation, we first LD pruned the markers in plink (indep-pairwise 50 50 0.5) and selected 50,000 random markers. PCA was estimated in plink, and the top three components were used as covariates in the mixed model to control for population structure. The best model was evaluated using Quantile–Quantile plots of the observed versus expected –log_10_ *P* values, which should follow a uniform distribution under the null hypothesis. SNPs with *P* values less than Bonferroni correction were considered significant. Genes within 100 Kbp of a significant marker were also considered significant.

### Signatures of selection and local adaptation

We employed two statistics for scanning the genome-wide data for signs of positive natural selection: iHS^56^ and Tajima’s D^57^. iHS is based on comparing the extended haplotype homozygosity score of the ancestral and derived allele of each marker. This test detects loci where natural selection is driving one haplotype to high frequency, leaving recombination little time to break up the linkage group. This was calculated using hapbin^58^. To find genomic regions associated with natural selection, we estimated the fraction of SNPs that have |iHS | >2.0 in each 100-Kbp window, with a slide of 10 Kbp. The windows with the highest fraction are considered outliers. Tajima’s D compares the average number of pairwise differences (π) and the number of segregating sites (S). A negative value indicates positive selection. These values were calculated for 10,000 windows (with a slide of 10,000), and the bottom 1% of the windows were considered outliers.

### GO and KEGG pathway enrichment analysis

GO enrichment analysis of positively selected genes was performed using topGO, an R Bioconductor package, to determine over-represented GO categories across biological process, cellular component and molecular function domains^59^. Enrichment of GO terms was tested using the ‘classic’ algorithm and two-sided Fisher’s exact test with *P* < 0.05 considered significant. KEGG^60^ pathway enrichment analysis was also performed on those gene sets based on a hypergeometric distribution test, and pathways with *P* < 0.05 were considered enriched. No adjustments were made for multiple tests.

### GWAS and validation of *SvLes1*

The GWAS population to assess seed shattering was planted in the greenhouse facility at the Donald Danforth Plant Science Center in April 2014. Two hundred fifteen accessions were chosen from the panel to perform the experiment (Supplementary Table 8), with four replicates per accession. Shattering phenotype was measured by observing the amount of seed shattering after hand shaking of senesced dry plants. Individual plants were scored using a qualitative scale from 1 to 7. Genotypes were filtered at MAF > 5% for this population. A GWAS was performed using a univariate mixed linear model from GEMMA^40^, with centered kinship matrix. We used the Wald test *P* value to assess significant peaks, but other *P-*value estimates give similar results. SNP effects were identified using snpEff^52^. Deleterious effects of missense SNPs were predicted using PROVEAN^26^ on both the reference and alternative allele against the National Center of Biotechnology Information nr protein database.

To knock out *SvLes1*, we used the backbone pTRANS_250d as described in ref. ^61^. The protospacer of the guide RNAs targeted the first and second exons of *SvLes1*, upstream of the predicted causal mutation, to ensure knockout by frameshift (Supplementary Fig. 12a). The binary vector was introduced into callus tissue using AGL1 agrobacterium. Tissue culture and transformation followed an established protocol for *S. viridis*^62^. T0 and T1 individuals were genotyped to identify newly acquired mutations near the targeted sites. A T2 homozygote *SvLes1-CRISPR1* was obtained and confirmed by Sanger sequencing, together with homozygotes of the unedited reference line for comparison. To test whether the non-shattering phenotype could be attributed to a single gene, we grew out the T3 seed from two presumptive T2 heterozygotes and several presumptive homozygotes and assessed their genotype at *SvLes1* by PCR and sequencing. Three inflorescences per plant were bagged at heading, and the bags were left on until about half the seeds in the panicle appeared mature. Seeds that had fallen off in the bag were collected and weighed. Weights fell largely into two categories, either less than 35 mg or more than 195 mg, with only a few weights in between, consistent with the effect of a single gene.

### Germination rate, seed weight and dispersal distance

Seeds from each genotype were collected, incubated at −80 °C overnight and then chlorine gas sterilized for 4 h in a bell jar. Glumes were manually removed, and seeds were sowed onto 0.5× MS with 1% sucrose plates and incubated in the dark at 4 °C for 2 d. Plates were moved into a growth chamber (12 h light (156 µmol m^−2^ s^−^^1^) / 12 h dark, 31 °C days and 22 °C nights), and percent germination (judged by the emerging radicle tip) was recorded every 24 h.

Seeds were harvested and pooled from five independent plants of each genotype. Five independent replicates of 20 seeds were weighed and recorded.

Starting at 1 week after heading and continuing to maturity, four random plants, each from each of two sibling families of *SvLes1-CRISPR1* and wild-type *SvLes1-1* (ME034V), were individually put into a specially designed pot holder in a wind tunnel custom built for the Kellogg Lab with seed collection bins approximately every 10 cm. Blower speed was set to 5 m.p.h. for 5 min. Seeds collected in bins at various distances were counted to measure dispersal distance from the parent plant.

### Tensile strength measurement

Seeds of *SvLes1-1* (ME034v), *SvLes1-2* (A10.1) and *SvLes1-CRISPR1* were treated with 5% liquid smoke overnight at room temperature and kept in wet moss at 4 °C in the dark for 2–3 weeks. Seeds were sown in Metro Mix 360 and grown in a greenhouse with a 14-h light/10-h dark cycle, day and night temperatures of 28 °C and 22 °C, respectively, and relative humidity of 40–50%. Panicles from main stems were collected at 8, 10, 12, 14, 16, 18, 20, 24 and 29 d after heading (the apex of the panicles emerged from the leaf sheath). Tensile strength of the spikelet and pedicel junction was measured as described previously^27^. Briefly, panicles were hung upside down from a Mark-10 model M3-2 force gauge. Spikelets were pulled off individually from a panicle using forceps, and the peak tension was recorded. Only the most developed spikelets from the central third of the panicle were used, to minimize the effects of developmental variation of the spikelets. Three plants with 20 spikelets from each plant were used per genotype per day of measurement. For *SvLes1-1* and *SvLes1-CRISPR1*, the plants in each genotype were offspring of two individual parent plants with the same allele.

### Histology

Histological procedures followed ref. ^63^. Specifically, primary branches were collected from the central third of panicles 12 and 16 d after heading and fixed in FAA (37% formaldehyde: ethanol: H_2_O: acetic acid = 10:50:35:5), followed by a dehydration series in 50%, 70%, 85%, 95%, 100%, 100% and 100% ethanol and 25%, 50%, 75%, 100%, 100% and 100% Histo-Clear (National Diagnostics) series with ethanol as solvent. Paraplast (Leica Biosystems) was then added to each vial of samples and kept overnight, heated at 42 °C and placed in a 60 °C oven. The solution was replaced with molten Paraplast twice a day for 3 d. Samples were then embedded in paraffin using a Leica EG1150 tissue embedder, sectioned in 10-µm serial slices with a Leica RM2255 automated microtome and mounted on microscope slides at 42 °C on a Premiere XH-2001 Slide Warmer. Sections were then deparaffinized, rehydrated, stained with 0.05% (wt/vol) toluidine blue O for 1.5 min and then rinsed with water, dehydrated in ethanol, cleared with xylene and mounted with Permount Mounting Medium (Electron Microscopy Sciences). Images were taken using a Leica DM750 LED Biological Microscope with ICC50 camera module and Leica Acquire version 2.0 software. Experiments were repeated on three independent plants of each genotype.

### Domestication selective sweep

Raw sequencing reads of foxtail millet lines were obtained from a previous study^32^. Because the average sequencing coverage in the earlier study (~0.5×) was much lower than in our study, we chose 79 lines (Supplementary Table 16) that have an estimated coverage >1× to maximize overlapping SNPs and perform analysis. Briefly, *S. italica* sequences were quality trimmed using sickle (https://github.com/najoshi/sickle) and aligned with bwa-mem to our *S. viridis* A10.1 genome. Multi-sample SNP calling was performed using samtools and Varscan with a minimum depth of 3. For *S. viridis*, the imputed, phased vcf was used for calculation of π, which uses high coverage. π calculation excluded missing samples. Shared SNPs between *S. italica* and *S. viridis* were combined, and missing data were imputed using Beagle 5.0 (ref. ^64^). Nucleotide diversity values πviridis and πitalica were then calculated using vcftools at 100-kb window size. Using genome-wide nucleotide diversity as a reference, we used the program ms^65^ to conduct 100,000 coalescent simulations to estimate the variation range of πitalica/πviridis under a domestication bottleneck model for a window of 20 kb. Strength of the bottleneck was determined by genome-wide πitalica/πviridis. The estimated ranges were then compared to observed values πitalica/πviridis to determine significance of domestication selective sweep regions.

### Retrotransposon insertion in *S. italica Les1*

*Copia38* sequence was obtained from the foxtail millet genomic sequence^66^ near the ortholog of *SvLes1*, Seita.5G087200 (Si003873m.g). We confirmed the identity of *Copia38* and identified its LTR region by searching its sequence against repbase (https://www.girinst.org/repbase/). We used National Center of Biotechnology Information blastn to identify close homologs of *Copia38* in the Yugu1 (ref. ^66^) and A10.1 genomes. RaxML 8.2.9 (ref. ^67^) was used to construct the phylogeny of *Copia38* homologs, and pairwise distances of close homologs to *Copia38* were calculated using Kimura 2 parameter model. Read mapping to Yugu1 genome follows similar procedures described previously. PE reads spanning beyond the left and right junction point of *Copia38* were used to determine whether the insertion occurs in an accession (Supplementary Fig. 12b).

### BSA mapping for small leaf angle

The cross between TB159 and A10.1 used pollen of TB159 and follows the protocol described in ref. ^68^. F_1_ individuals were naturally self-pollinated to generate an F_2_ population. Four hundred seventeen F_2_ individuals were planted and phenotypically scored, and DNA from 30 small leaf angle individuals was pooled and sequenced. Sequences are available in the Sequence Read Archive at the National Center for Biotechnology Information, BioProject number PRJNA527194. The analysis follows the methods described in a previous BSA study in *S. viridis*^20^. Identification of disruptive mutations and missense mutations with deleterious effects follows the same approach described in our GWAS study. Syntenic orthology between *SvLg2* and *liguleless2* in maize was examined and confirmed based on ref. ^69^.

### Online statistics section

To test which genes were significantly over- or under-represented within each of the four *S. viridis* subpopulations (‘Central’, ‘Central-East’, ‘Central-North’ and ‘West-Coast’), a χ^2^ test was performed on each of the 51,323 genes within the pan-genome. For each gene, the total number of observations across each subpopulation was counted (observed gene count). The expected gene count per subpopulation was calculated from the total observations multiplied by the proportion of individuals within each subpopulation (*n* = 35, 59, 78 and 130, respectively; total number of individuals = 302). A χ^2^ test (degrees of freedom = 3; *P* value = 0.05; critical value = 7.81) was performed on each gene, retaining whose χ^2^ value was greater than the critical value. χ^2^ values were converted to *P* values, and a Benjamini–Hochberg correction was performed to correct for false discoveries.

GO enrichment analysis of positively selected genes was performed using topGO, an R Bioconductor package, to determine over-represented GO categories across biological process, cellular component and molecular function domains^59^. Enrichment of GO terms was tested using the ‘classic’ algorithm, and two-sided Fisher’s exact test with *P* < 0.05 was considered significant. KEGG^60^ pathway enrichment analysis was also performed on those gene sets based on a hypergeometric distribution test, and pathways with *P* < 0.05 were considered enriched. No adjustments were made for multiple tests.

### Reporting Summary

Further information on research design is available in the Nature Research Reporting Summary linked to this article.

## Online content

Any methods, additional references, Nature Research reporting summaries, source data, extended data, supplementary information, acknowledgements, peer review information; details of author contributions and competing interests; and statements of data and code availability are available at 10.1038/s41587-020-0681-2.

## Supplementary information

Supplementary Information Supplementary Figs. 1–12 and Supplementary Tables 2, 4 and 9–14

Reporting Summary

Supplementary Table 1 Metadata for 598 diversity lines.

Supplementary Table 3 PAV data for each subpopulation.

Supplementary Tables 5 and 6 Annotation of genes containing markers significantly associated with environmental variables.

Supplementary Table 7 KEGG pathways enriched for genes under selection by iHS.

Supplementary Table 8 Samples.scored for shattering.

Supplementary Table 15 PAV matrix, non-admixed samples.

Supplementary Table 16 Accession numbers for *italica* lines.

## Data Availability

Sequences are available in the Sequence Read Archive at the National Center of Biotechnology Information, BioProject numbers PRJNA560514 and PRJNA265547. Sequences for the bulk segregant analysis are in the BioProject PRJNA527194. The *Setaria viridis* A10.1 genome is SNSE00000000.1. Sequence Read Archive accession numbers for the individual diversity lines are in Supplementary Table 1. Seed stocks for diversity lines are available by contacting co-authors I.B. or E.A.K.

## References

[1] Kellogg, E. A. in *Families and Genera of Vascular Plants* (ed Kubitzki, K) 1–416 (Springer, 2015).

[2] Brutnell TP (2010). *Setaria viridis*: a model for C_4_ photosynthesis. Plant Cell.

[3] Henry C (2020). Sugar sensing responses to low and high light in leaves of the C_4_ model grass *Setaria viridis*. J. Exp. Bot..

[4] Saha P (2016). Effects of abiotic stress on physiological plasticity and water use of *Setaria viridis* (L.). Plant Sci..

[5] Ferreira SS (2019). The lignin toolbox of the model grass *Setaria viridis*. Plant Mol. Biol..

[6] Yang J (2018). Brassinosteroids modulate meristem fate and differentiation of unique inflorescence morphology in *Setaria viridis*. Plant Cell.

[7] Junqueira NEG (2018). Anatomy and ultrastructure of embryonic leaves of the C_4_ species *Setaria viridis*. Ann. Bot..

[8] Hunter CT (2020). *Setaria viridis* as a model for translational genetic studies of jasmonic acid-related insect defenses in *Zea mays*. Plant Sci..

[9] Rodríguez CE, Antonielli L, Mitter B, Trognitz F, Sessitsch A (2020). Heritability and functional importance of the *Setaria viridis* bacterial seed microbiome. Phytobiomes.

[10] Ribeiro, A. P. et al. Overexpression of *BdMATE* gene improves aluminum tolerance in *Setaria viridis*. *Front. Plant Sci.* **8**, 865 (2017). 10.3389/fpls.2017.00865 PMC5462932 28642761

[11] Dangol, A., Yaakov, B., Jander, G., Strickler, S. R. & Tzin, V. Characterizing the serotonin biosynthesis pathway upon aphid infestation in *Setaria viridis* leaves. Preprint at https://www.biorxiv.org/content/10.1101/642041v1 (2019). 10.1007/s11103-021-01239-4 35020104

[12] Liu, Y. et al. A biomimetic Setaria viridis-inspired electrode with polyaniline nanowire arrays aligned on MoO3@polypyrrole core–shell nanobelts. *J. Mater. Chem. A Mater*. **6**, 13428–13437 (2018).

[13] Yu, Y. & Kellogg, E. A. Inflorescence abscission zones in grasses: diversity and genetic regulation. *Annu. Plant Rev.*10.1002/9781119312994.apr0619 (2018).

[14] Fuller DQ (2007). Contrasting patterns in crop domestication and domestication rates: recent archaeobotanical insights from the Old World. Ann. Bot..

[15] Hunt HV (2008). Millets across Eurasia: chronology and context of early records of the genera *Panicum* and *Setaria* from archaeological sites in the Old World. Veg. Hist. Archaeobot..

[16] Kennard C, Phillips L, Porter A (2002). Genetic dissection of seed shattering, agronomic, and color traits in American wildrice (*Zizania palustris* var. *interior* L.) with a comparative map. Theor. Appl. Genet..

[17] Yu Y, Hu H, Doust AN, Kellogg EA (2020). Divergent gene expression networks underlie morphological diversity of abscission zones in grasses. New Phytol..

[18] Waterhouse, R. M. et al. BUSCO applications from quality assessments to gene prediction and phylogenomics. *Mol. Biol. Evol.* **35***,* 543–548 (2017). 10.1093/molbev/msx319 PMC5850278 29220515

[19] Layton DJ, Kellogg EA (2014). Morphological, phylogenetic, and ecological diversity of the new model species *Setaria viridis* (Poaceae: Paniceae) and its close relatives. Amer. J. Bot..

[20] Huang P (2017). *Sparse panicle1* is required for inflorescence development in *Setaria viridis* and maize. Nat. Plants.

[21] Raj A, Stephens M, Pritchard J (2014). fastSTRUCTURE: variational inference of population structure in large SNP data sets. Genetics.

[22] Shomura A (2008). Deletion in a gene associated with grain size increased yields during rice domestication. Nat. Genet..

[23] Jombart T, Ahmed I (2011). adegenet 1.3-1: new tools for the analysis of genome-wide SNP data. Bioinformatics.

[24] Gordon SP (2017). Extensive gene content variation in the *Brachypodium distachyon* pan-genome correlates with population structure. Nat. Commun..

[25] Fick SE, Hijmans RJ (2017). WorldClim 2: new 1-km spatial resolution climate surfaces for global land areas. Int. J. Climatol..

[26] Choi Y, Chan AP (2015). PROVEAN web server: a tool to predict the functional effect of amino acid substitutions and indels. Bioinformatics.

[27] Hodge JG, Kellogg EA (2016). Abscission zone development in *Setaria viridis* and its domesticated relative, *Setaria italica*. Am. J. Bot..

[28] Li C, Zhou A, Sang T (2006). Rice domestication by reducing shattering. Science.

[29] Konishi S (2006). An SNP caused loss of seed shattering during rice domestication. Science.

[30] Zhou Y (2012). Genetic control of seed shattering in rice by the APETALA2 transcription factor *SHATTERING ABORTION1*. Plant Cell.

[31] Lin Z (2012). Parallel domestication of the *Shattering1* genes in cereals. Nat. Genet..

[32] Jia G (2013). A haplotype map of genomic variations and genome-wide association studies of agronomic traits in foxtail millet (*Setaria italica*). Nat. Genet..

[33] Odonkor S (2018). QTL mapping combined with comparative analyses identified candidate genes for reduced shattering in *Setaria italica*. Front. Plant Sci..

[34] Gaut BS, Morton BR, McCaig BC, Clegg MT (1996). Substitution rate comparisons between grasses and palms: synonymous rate differences at the nuclear gene *Adh* parallel rate differences at the plastid gene *rbcL*. Proc. Natl Acad. Sci. USA.

[35] Tian F (2011). Genome-wide association study of leaf architecture in the maize nested association mapping population. Nat. Genet..

[36] Walsh J, Waters CA, Freeling M (1998). The maize gene *liguleless2* encodes a basic leucine zipper protein involved in the establishment of the leaf blade-sheath boundary. Genes Dev..

[37] Thielen, P. M., et al. Reference genome for the highly transformable *Setaria viridis* cultivar ME034V. *G3 (Bethesda)* (in the press). 10.1534/g3.120.401345 PMC7534418 32694197

[38] Feldman MJ (2018). Components of water use efficiency have unique genetic signatures in the model C_4_ grass *Setaria*. Plant Physiol..

[39] Hellmann H, Estelle M (2002). Plant development: regulation by protein degradation. Science.

[40] Zhou X, Stephens M (2014). Efficient multivariate linear mixed model algorithms for genome-wide association studies. Nat. Methods.

[41] Wang ZM, Devos KM, Liu CJ, Wang RQ, Gale MD (1998). Construction of RFLP-based maps of foxtail millet, *Setaria italica* (L.) P. Beauv. Theor. Appl. Genet..

[42] Huang P (2014). Population genetics of *Setaria viridis*, a new model system. Mol. Ecol..

[43] Xiao CL (2017). MECAT: fast mapping, error correction, and de novo assembly for single-molecule sequencing reads. Nat. Methods.

[44] Chin CS (2013). Nonhybrid, finished microbial genome assemblies from long-read SMRT sequencing data. Nat. Methods.

[45] Li, H. Aligning sequence reads, clone sequences and assembly contigs with BWA-MEM. Preprint at https://arxiv.org/abs/1303.3997 (2013).

[46] McKenna A (2010). The Genome Analysis Toolkit: a MapReduce framework for analyzing next-generation DNA sequencing data. Genome Res..

[47] Krumsiek J, Arnold R, Rattei T (2007). Gepard: a rapid and sensitive tool for creating dotplots on genome scale. Bioinformatics.

[48] Lovell JT (2018). The genomic landscape of molecular responses to natural drought stress in *Panicum hallii*. Nat. Commun..

[49] Haas BJ (2003). Improving the Arabidopsis genome annotation using maximal transcript alignment assemblies. Nucleic Acids Res..

[50] Chapman, J. A. Meraculous2: fast accurate short-read assembly of large polymorphic genomes. Preprint at https://arxiv.org/abs/1608.01031 (2016).

[51] Koboldt DC (2012). VarScan 2: somatic mutation and copy number alteration discovery in cancer by exome sequencing. Genome Res..

[52] Cingolani P (2012). A program for annotating and predicting the effects of single nucleotide polymorphisms, SnpEff: SNPs in the genome of *Drosophila melanogaster* strain w1118; iso-2; iso-3. Fly (Austin).

[53] Sonnhammer EL, Ostlund G (2015). InParanoid 8: orthology analysis between 273 proteomes, mostly eukaryotic. Nucleic Acids Res..

[54] Sedlazeck FJ (2018). Accurate detection of complex structural variations using single-molecule sequencing. Nat. Methods.

[55] Chang CC (2015). Second-generation PLINK: rising to the challenge of larger and richer datasets. Gigascience.

[56] Voight BF, Kudaravalli S, Wen X, Pritchard JK (2006). A map of recent positive selection in the human genome. PLoS Biol..

[57] Tajima F (1989). Statistical method for testing the neutral mutation hypothesis by DNA polymorphism. Genetics.

[58] Maclean CA, Chue Hong NP, Prendergast JG (2015). hapbin: an efficient program for performing haplotype-based scans for positive selection in large genomic datasets. Mol. Biol. Evol..

[59] Alexa A, Rahnenfuhrer J, Lengauer T (2006). Improved scoring of functional groups from gene expression data by decorrelating GO graph structure. Bioinformatics.

[60] Kanehisa M, Goto S (2000). KEGG: Kyoto encyclopedia of genes and genomes. Nucleic Acids Res..

[61] Cermák T (2017). A multipurpose toolkit to enable advanced genome engineering in plants. Plant Cell.

[62] VanEck, J., Swartwood, K., Pidgeon, K. & Maxon-Stein, K. in *Genetics and Genomics of* Setaria *Plant Genetics and Genomics: Crops and Models* (eds Doust, A. & Diao, X.) 343–356 (Springer, 2016).

[63] Ruzin, S. E. *Plant Microtechnique and Microscopy* (Oxford University Press, 1999).

[64] Browning BL, Browning SR (2016). Genotype imputation with millions of reference samples. Am. J. Hum. Genet..

[65] Hudson RR (2002). Generating samples under a Wright–Fisher neutral model of genetic variation. Bioinformatics.

[66] Bennetzen JL (2012). Reference genome sequence of the model plant *Setaria*. Nat. Biotechnol..

[67] Stamatakis A (2014). RAxML version 8: a tool for phylogenetic analysis and post-analysis of large phylogenies. Bioinformatics.

[68] Jiang, H., Barbier, H. & Brutnell, T. Methods for performing crosses in *Setaria viridis*, a new model system for the grasses. *J. Vis. Exp.*10.3791/50527 (2013). 10.3791/50527 PMC3938206 24121645

[69] Schnable JC, Freeling M, Lyons E (2012). Genome-wide analysis of syntenic gene deletion in the grasses. Genome Biol. Evol..

