# Supplementary material for: A genome resource for green millet Setaria viridis enables discovery of agronomically valuable loci
Source: Nat Biotechnol. 2020 Oct 5;38(10):1203–10. doi: 10.1038/s41587-020-0681-2 (PMC7536120; doi:10.1038/s41587-020-0681-2)
Supplement: Supplementary file 1 — Supplementary Figs. 1–12 and Supplementary Tables 2, 4 and 9–14 [file 41587_2020_681_MOESM1_ESM.pdf]

natureresearch
https://doi.org/10.1038/s41587-020-0681-2

Supplementary information

A genome resource for green millet *Setaria viridis* enables discovery of agronomically valuable loci

In the format provided by the authors and unedited

Supplementary Figure 1. Cumulative citations for *Setaria viridis*, from Scopus, accessed Jan. 2, 2020.

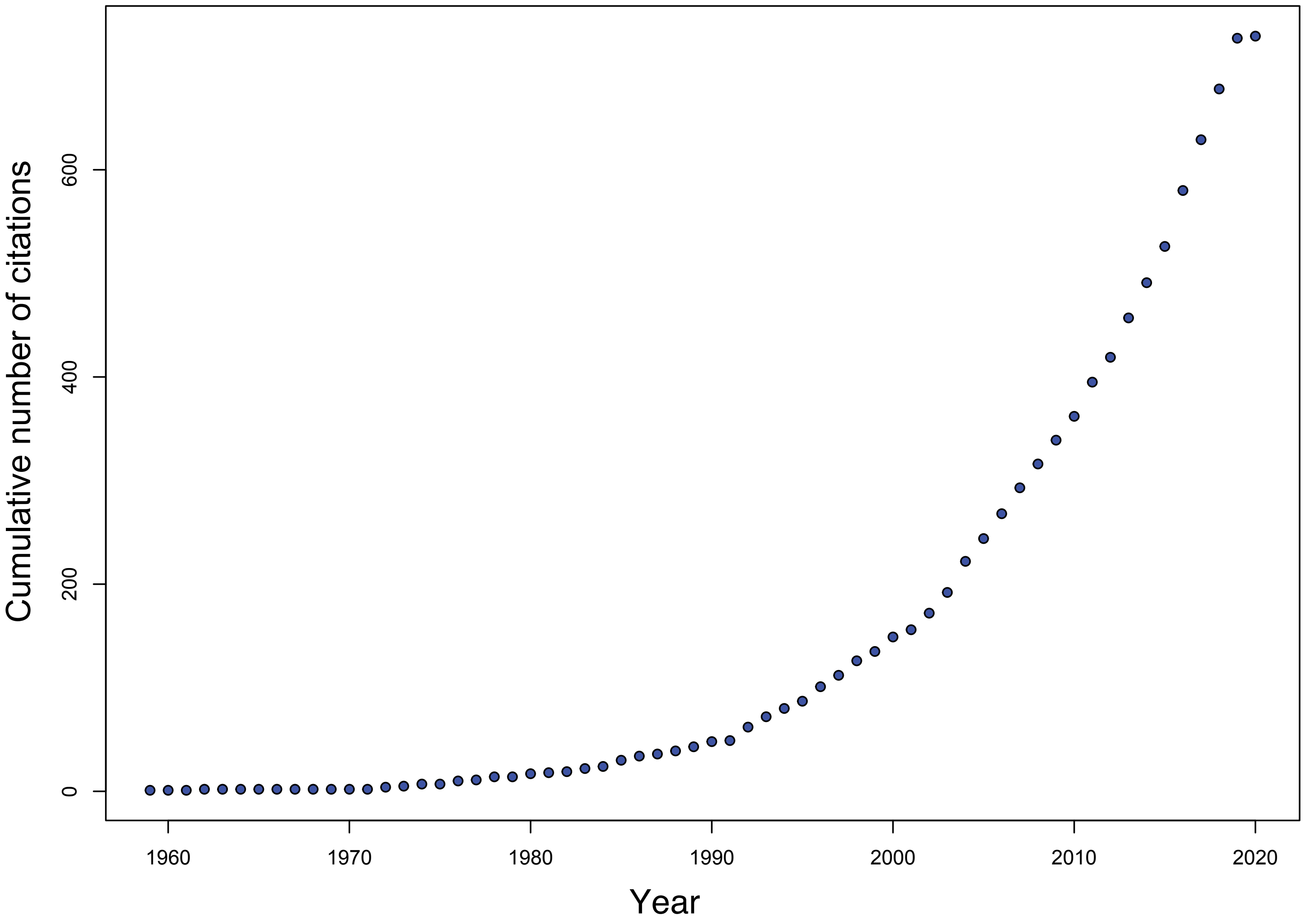

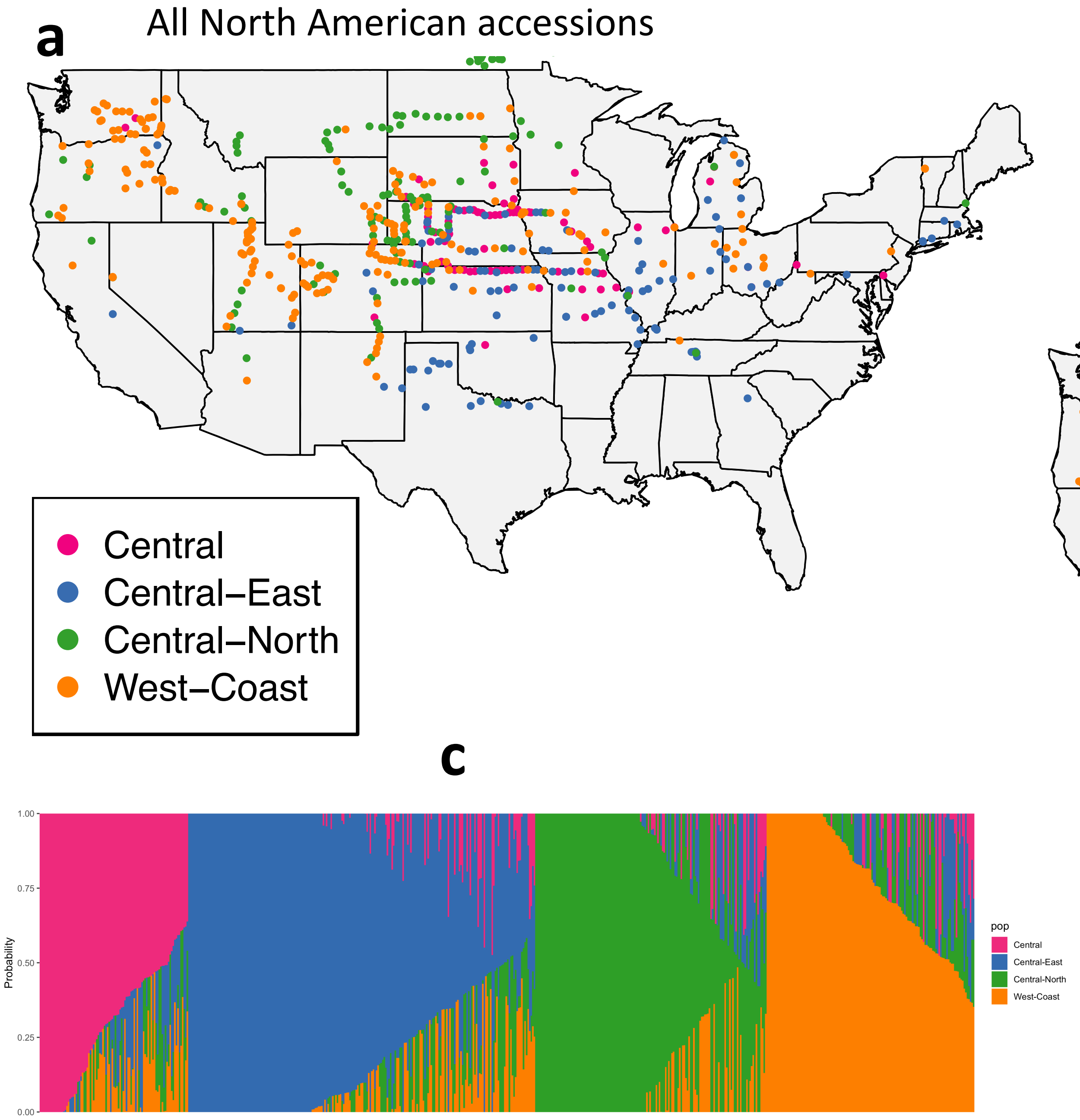

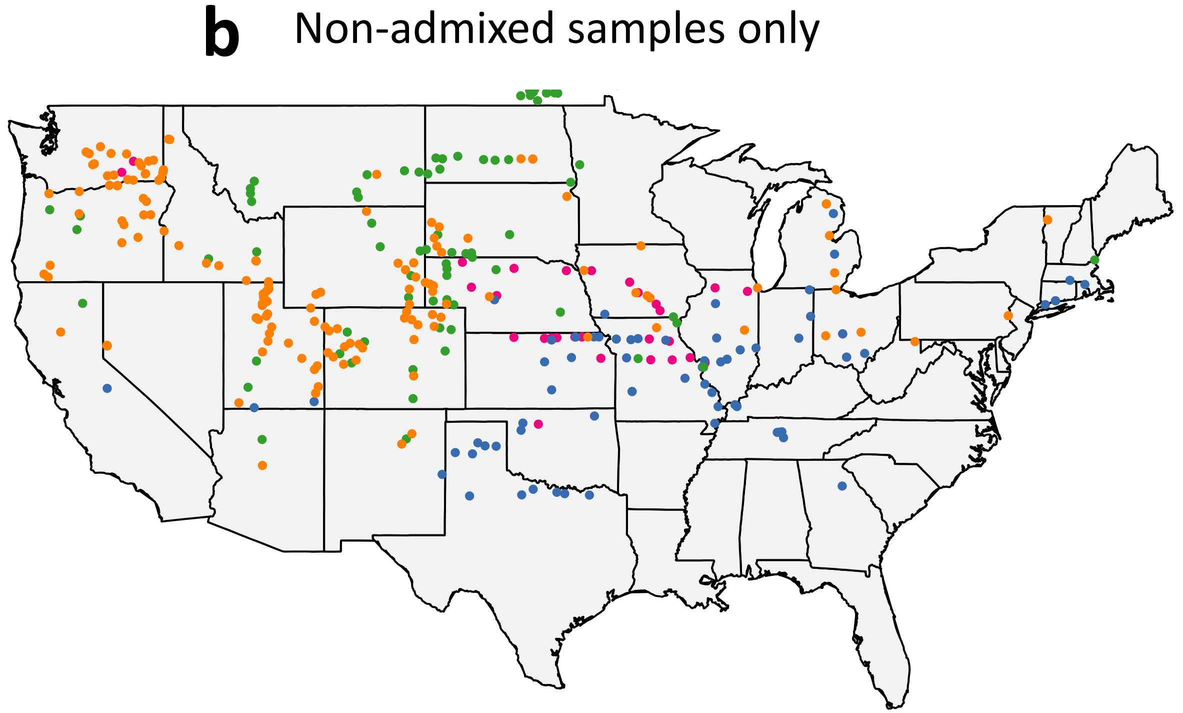

Supplementary Figure 2. Population distribution and structure of North American accessions. Population assignment based on SNPs. **a**, distribution of all accessions; **b**, distribution of non-admixed samples only; **c**, STRUCTURE plot including all accessions.

Supplementary Figure 3. Over-represented GO categories and KEGG enrichment pathways for each subpopulation. Enrichment of GO terms was tested using the “classic” algorithm and two-sided Fisher's exact test with $p<0.05$ considered as significant. KEGG pathway enrichment analysis was based on a hypergeometric distribution test and pathways with $p<0.05$ were considered as enriched. No adjustments were made for multiple tests.

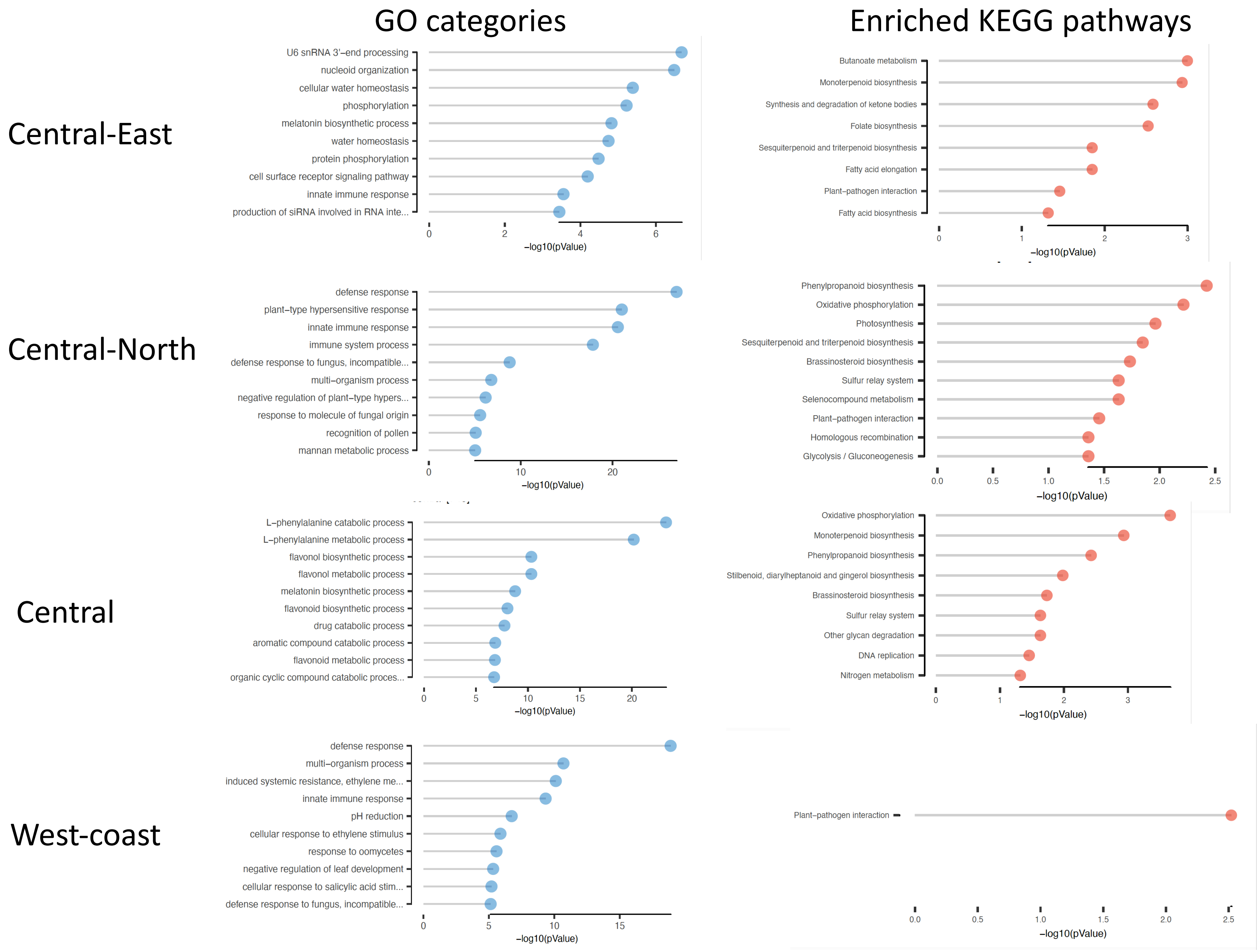

Supplementary Figure 4. Correlations among the bioclim variables for the 577 samples with lat-long coordinates.

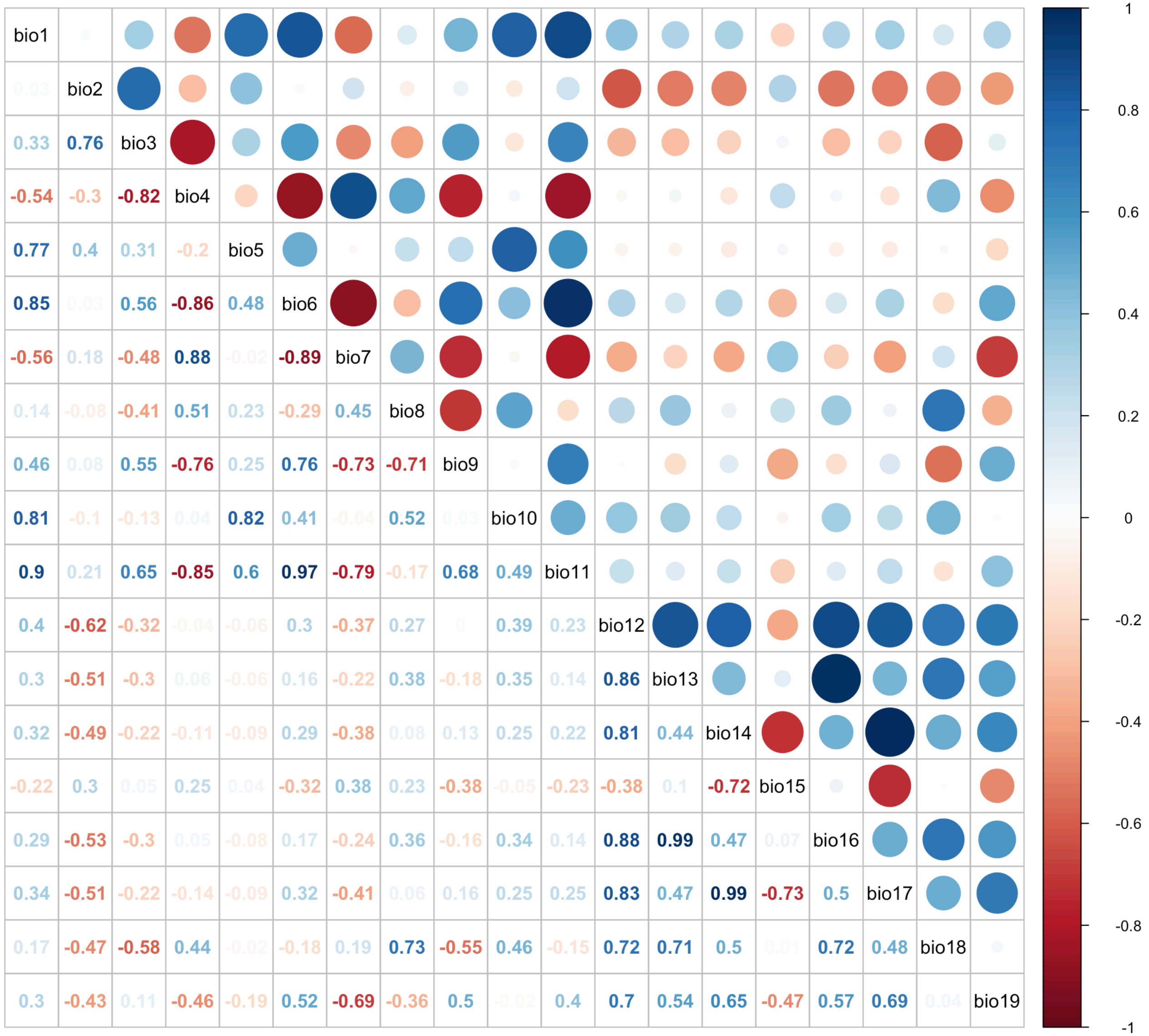

Supplementary Figure 5. GWAS results using each of the first three environmental PCs as response variable. PC1 found no significant loci, PC2 found one, and PC3 found several.

PC1

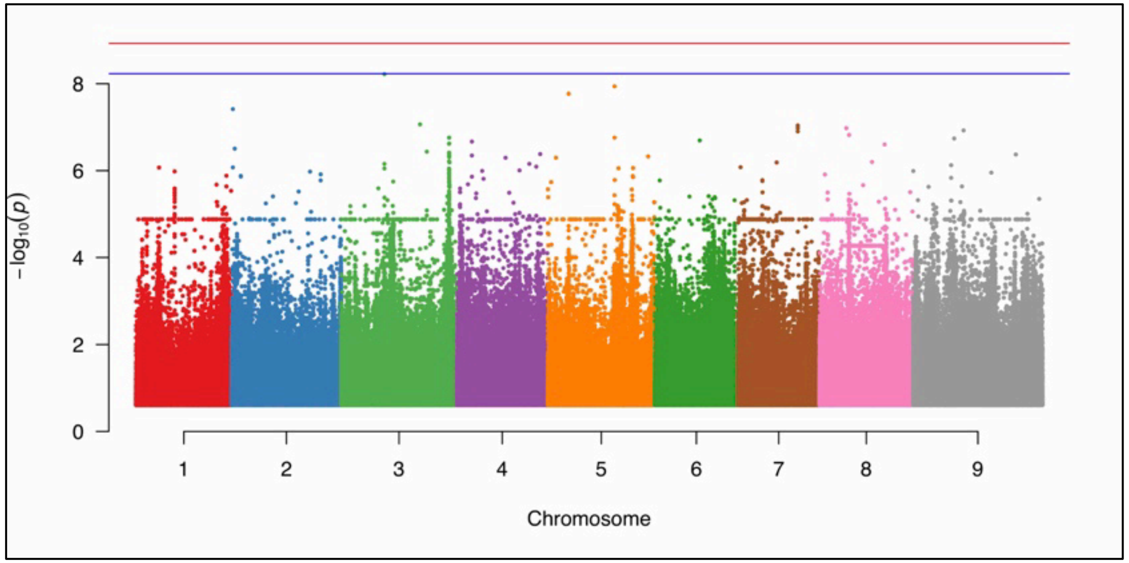

PC2

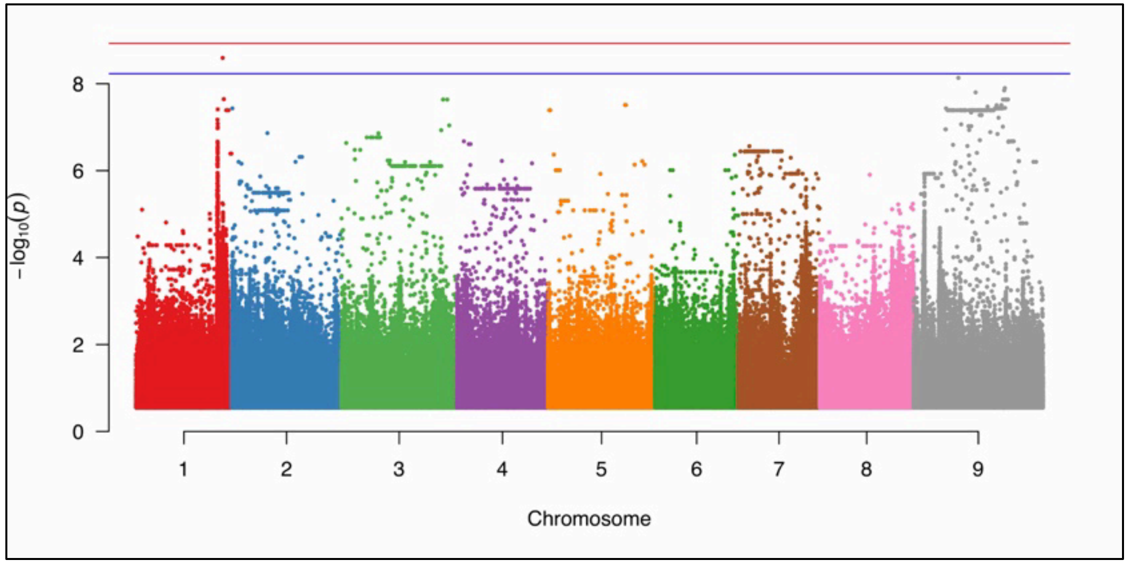

PC3

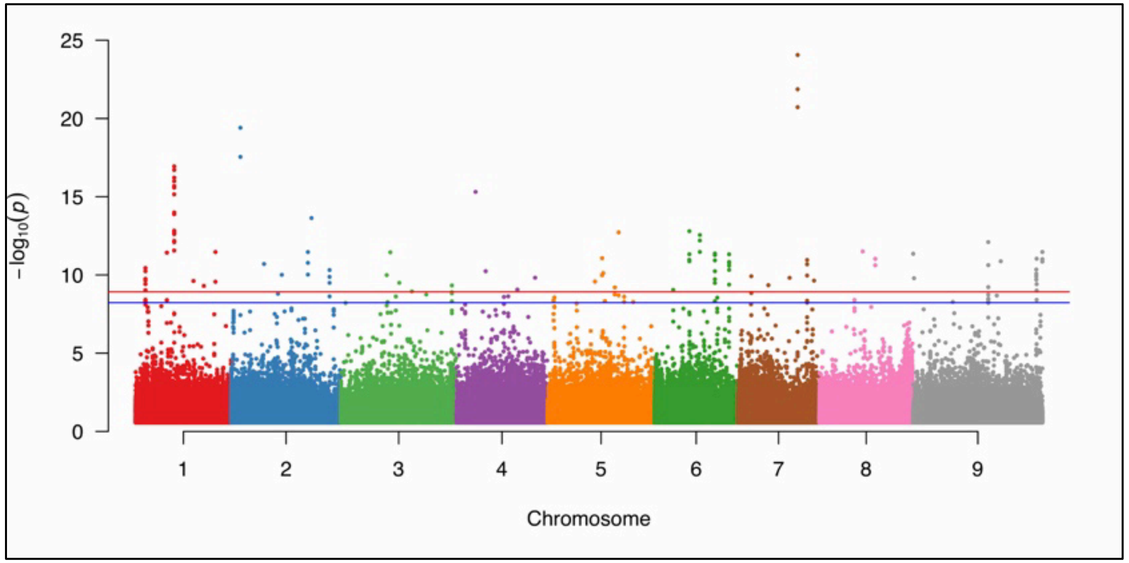

Supplementary Figure 6. Loci showing significant signatures of selection for each population. Left column, proportion of SNP with |iHS| >2 for each of the population. Right column, Tajima's D represented in -ve scale. 100 Kbp windows (slide 10 Kbp). Horizontal lines represent 95th and 99th percentiles for iHS and 1st and 5th percentiles for Tajima's D.

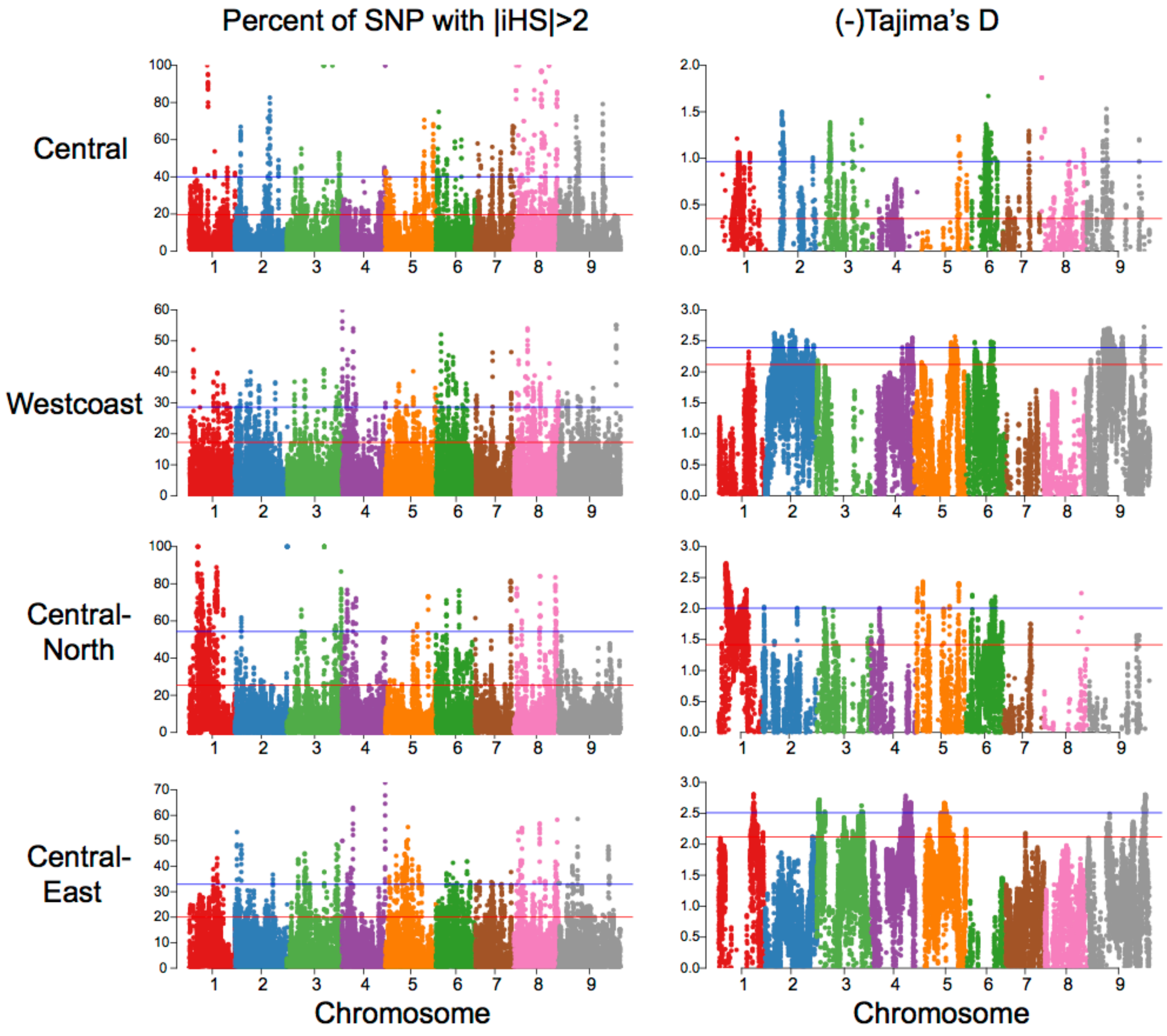

Supplementary Figure 7. a Comparison of seed germination rates of *SvLes1-1* and *SvLes1-CRISPR1* lines. Seeds were divided among four plates for each genotype (technical replicates), percent germination calculated for each plate at each time point, and then averaged within genotype. The experiment was repeated three times (biological replicates) with 83, 55, and 95 seeds of *SvLes1-1* (WT) for the three experiments, and 163, 112, 147 seeds of *SvLes1-CRISPR*. Black horizontal line is median, hinges are first and third quartiles, whiskers are 1.5 times the interquartile range, filled circles are outliers. **b**, Comparison of seed weight of *SvLes1-1* and *SvLes1-CRISPR1* lines. Twenty-seed weight was measured from 5 independent plants of *SvLes1-1* and 10 of *SvLes1-CRISPR1*. *SvLes1-CRISPR1* seeds weighed significantly less (p = 3.22E-05; 2-tailed T-test for 2 samples, unequal variances), but this did not affect germination. Error bars and symbols as in 7a.

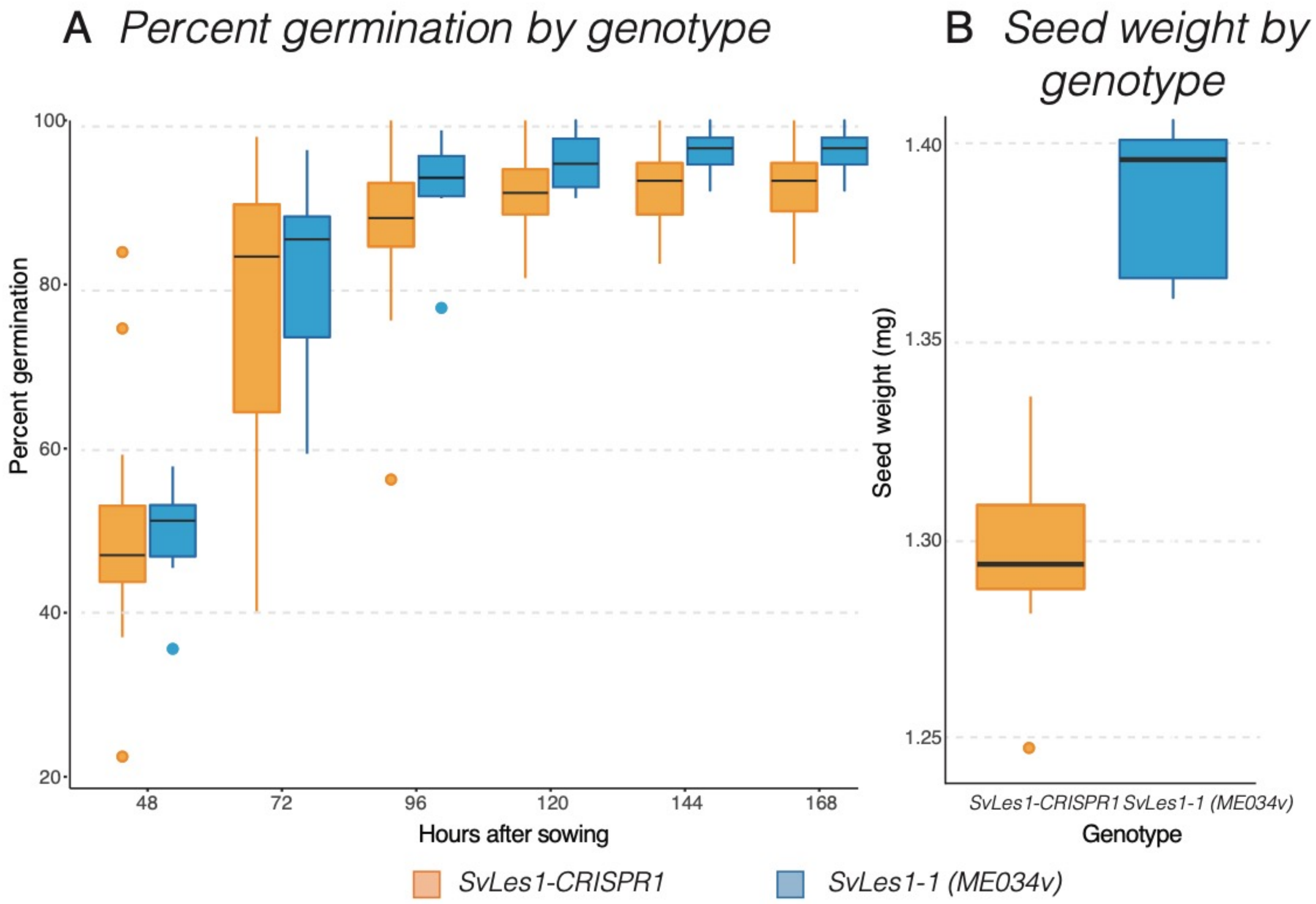

Supplementary Figure 8. *SvLes1-2* (A10.1), *SvLes1-1*, and *SvLes1-CRISPR1* have similar anatomical structures in the abscission zone. Spikelets from main panicles 12 days after heading, stained with 0.05% Toluidine blue O. **a,b** *SvLes1-2* (A10.1). **c,d** *SvLes1-1.* **e,f** *SvLes1-CRISPR1.* **a,c,e** Scale bars = 100 µm. **b,d,f** Scale bars **=** 20 µm. En, endosperm; em, embryo. White dotted line, approximate position of AZ. Experiments were repeated on three independent plants of each genotype.

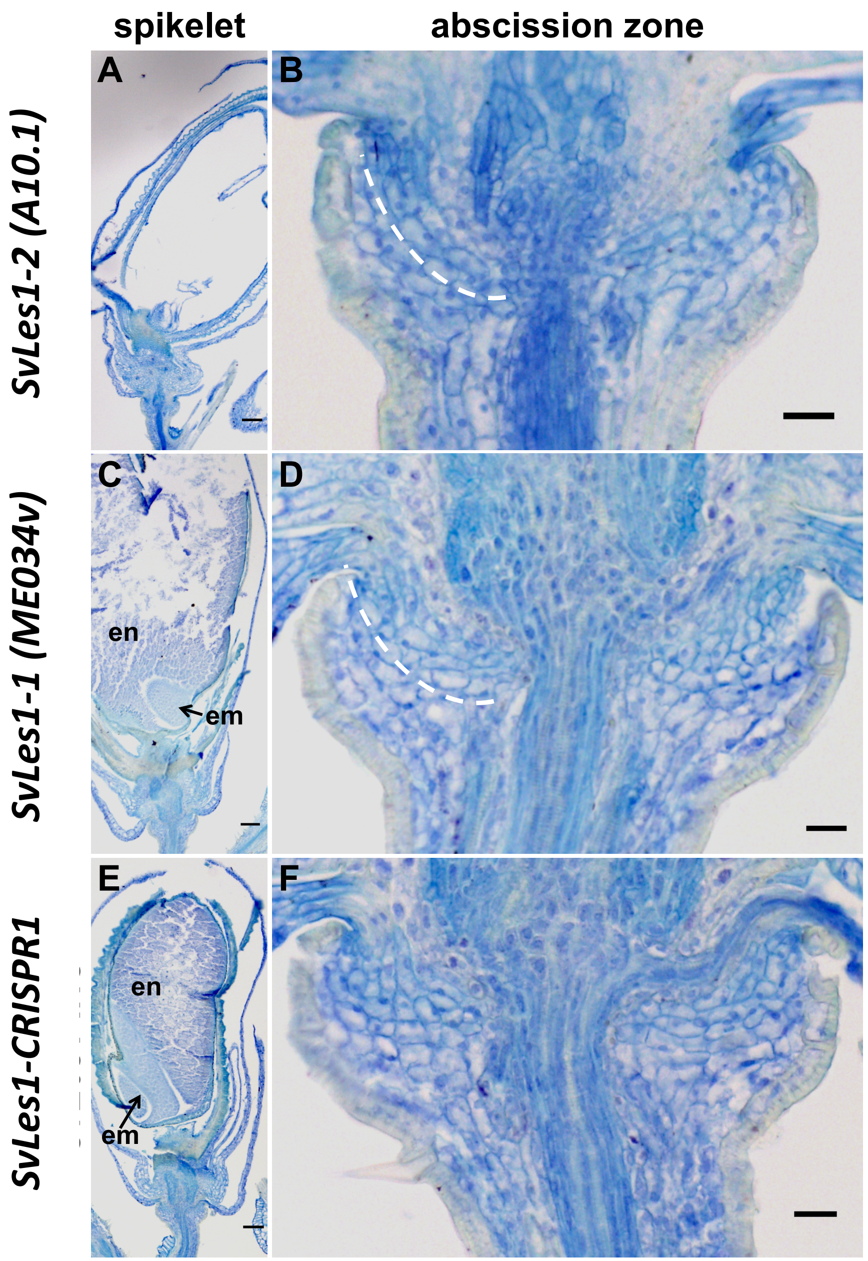

Supplementary Figure 9. Phylogenetic tree of *Copia38* copies in A10.1 and Yugu1 genomes. Red clade shows the recent explosion that contains the copy inserted into *SiLES1*. Gene models in green, *S. italica*; in blue, *S. viridis*.

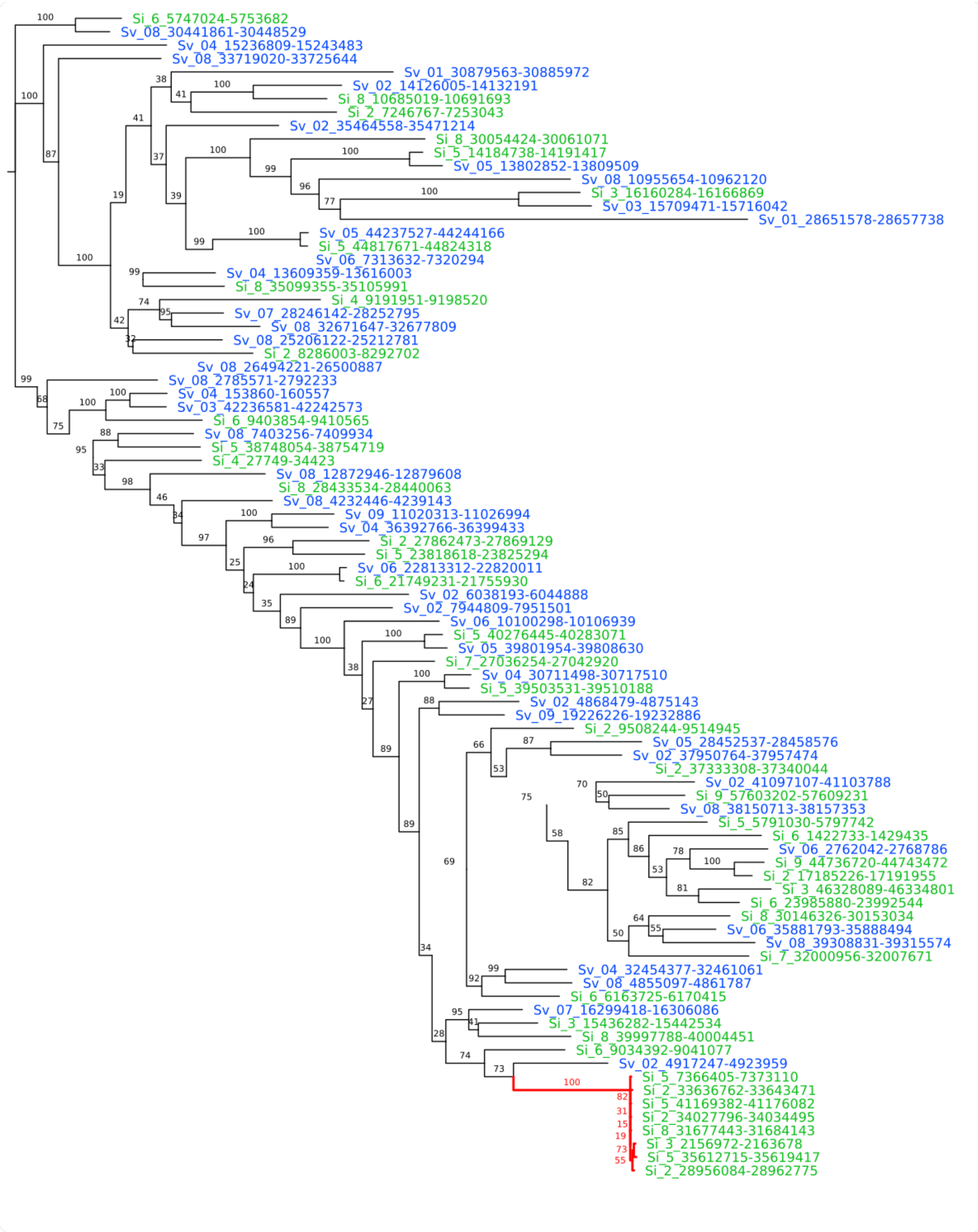

Supplementary Figure 10. Representive dot plots**. a** Dot plot of clone 114809 on a region of scaffold_1. This alignment is representative of the high quality clone alignments in 239 of the 365 available clones. **b** Dot plot of clone 113526 on a region of scaffold_3, which is representative of the 36 clones that landed in repetitive regions of the genome.

a

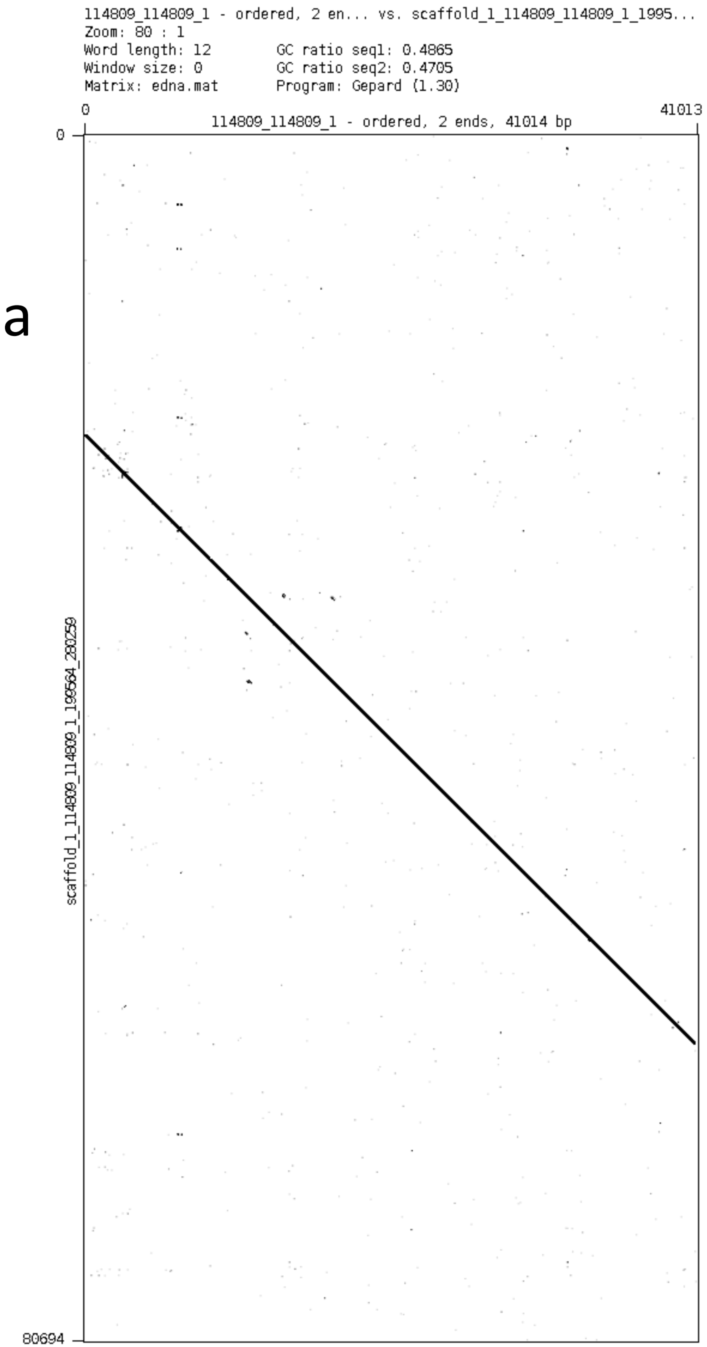

b

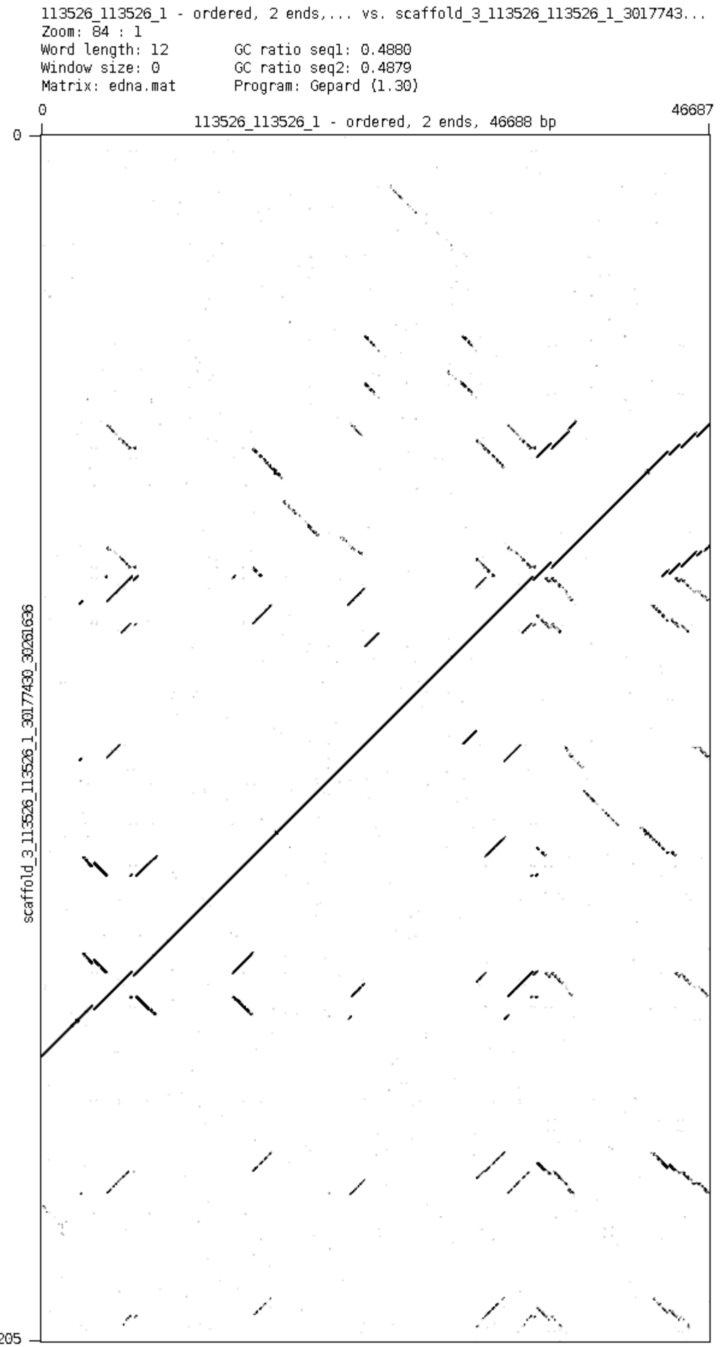

Supplementary Figure 11. Plots of marker placements for each of the 9 chromosomes (scaffolds) of *Setaria viridis*.

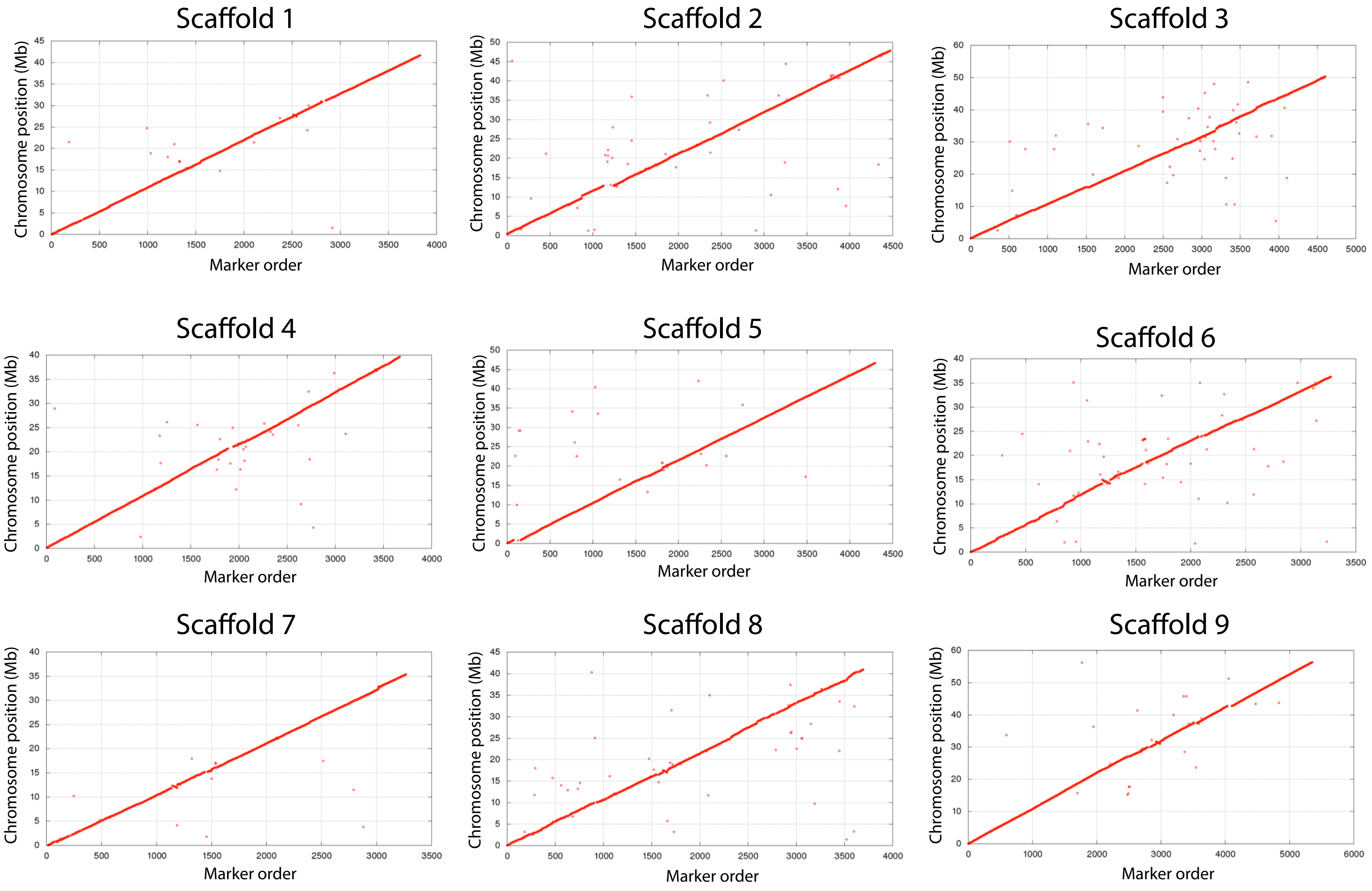

Supplementary Figure 12. a guide RNA protospacers and their position relative to allelic mutations and gene model. **b** Method used to detect *Copia38* insertion.

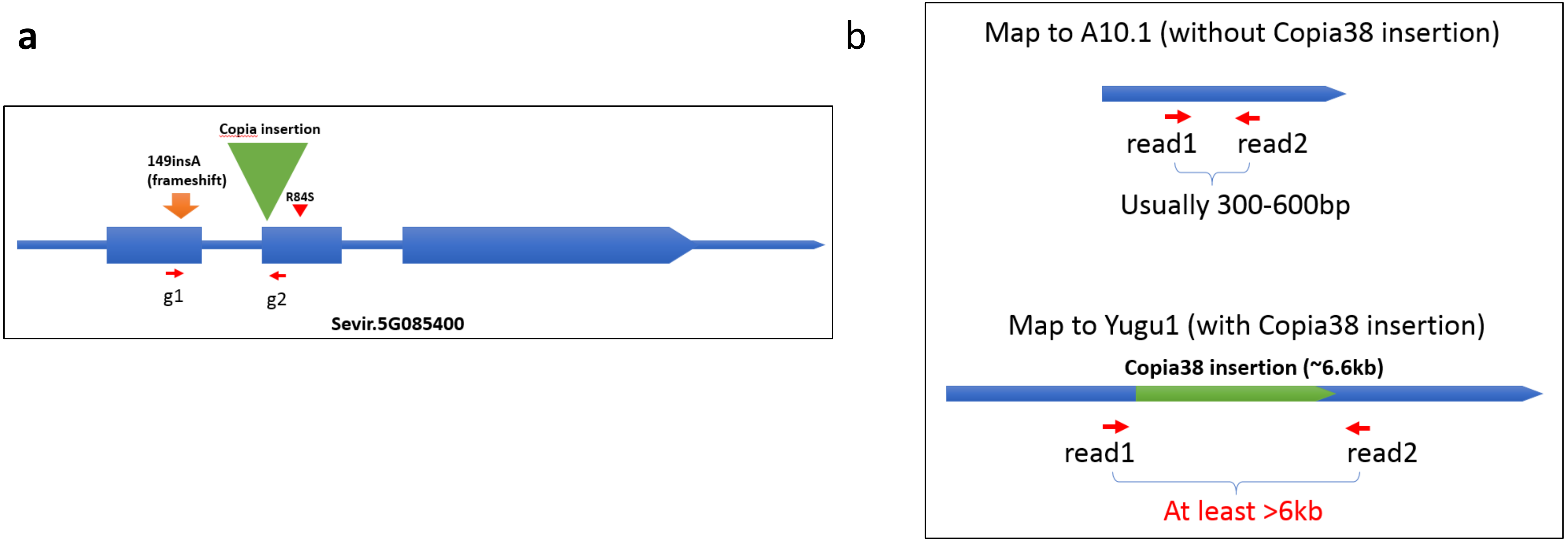

Supplementary Table 2. Subpopulation statistics. Numbers of non-admixed individuals based on SNP analysis; numbers used for each subpopulation in the PAV analysis are lower because not all libraries assembled well enough to be used. Number of over-represented genes is the number significant for that subpopulation alone. SNP, single nucleotide polymorphism; Pi/bp, polymorphisms per basepair; IBD, identity by descent for the subpopulation; IBS, identity by state for the subpopulation; LD, linkage disequilibrium; LD R^2>=0.7%, percent of pairwise comparisons with LD>=0.7.

Subpopulation	Central	Central East	Central North	Westcoast
# non-admixed individuals	44	81	104	153
Unique SNPs	836,585	21,747	368,608	183,712
Pi/bp	0.00457657	0.00235105	0.00373323	0.00259107
#over-represented genes	748	482	1904	435
Subpopulation-specific private alleles	24	0	20	1
IBD - Average (kb)	55.5021427	138.839981	162.239224	129.683235
IBS_average	8.2458E-05	1.6934E-05	2.3011E-05	1.998E-05
LD mean	0.2544468	0.4378058	0.3899508	0.4481402

(LD R^2>=0.7) %	0.167	0.359	0.306	0.365

Supplementary Table 4. Loadings of bioclim variables on the first three principal components. PC, principal component; min, minimum; max, maximum.

Variable code	Variable name			
		PC1	PC2	PC3
bio6	Min temperature warmest month	-0.326	-0.191	-0.089
bio11	Mean temperature coldest quarter	-0.305	-0.204	-0.185
bio1	Annual mean temperature	-0.300	-0.072	-0.324
bio19	Precipitation coldest quarter	-0.299	0.047	0.268
bio12	Annual precipitation	-0.278	0.276	0.037
bio17	Precipitation driest quarter	-0.277	0.191	0.162
bio14	Precipitation driest month	-0.264	0.194	0.155
bio9	Mean temperature driest quarter	-0.223	-0.273	0.127
bio16	Precipitation wettest quarter	-0.203	0.281	-0.049
bio13	Precipitation wettest month	-0.196	0.279	-0.067
bio10	Mean temperature warmest quarter	-0.173	0.092	-0.448
bio5	Max temperature warmest month	-0.108	-0.127	-0.468
bio3	Isothermality	-0.094	-0.349	-0.057
bio18	Precipitation warmest quarter	-0.078	0.364	-0.168
bio8	Mean temperature wettest quarter	0.048	0.260	-0.361
bio2	Mean diurnal temperature range	0.112	-0.283	-0.206
bio15	Precipitation seasonality	0.196	-0.010	-0.240
bio4	Temperature seasonality	0.245	0.288	-0.058
bio7	Temperature annual range	0.315	0.151	-0.146

Supplementary Table 9. Comparison of pairwise diversity in the region surrounding *SiLes1* vs. *SvLes1*. 100,000 coalescent simulations were conducted for πitalica/πviridis under a domestication bottleneck model for a window of 20 kb centered on the gene. Strength of the bottleneck was determined by genome wide πitalica/πviridis. Estimated values in the simulation were then compared to observed values πitalica/πviridis. The observed values for πitalica/πviridis were well below most values for the simulated data, with *p*=0.0066.

Description	Start	End	Length (bp)	π *italica*	π *viridis*	*italica* SNP	*viridis* SNP	π *italica*/ π *viridis*
Gene	6847970	6850236	2266	1.2650	4.4274	5	21	0.2857
10kb total	6844103	6854103	10000	3.2352	50.1570	96	189	0.0645
20kb total	6839103	6859103	20000	6.3833	135.5472	245	460	0.0471
50kb total	6824103	6874103	50000	20.9668	286.5908	717	1001	0.0732
10kb either side	6837970	6860236	22266	7.0845	159.3458	300	545	0.0445
20kb either side	6827970	6870236	42266	20.3942	247.2280	640	871	0.0825
50kb either side	6797970	6900236	102266	39.1945	959.0633	1129	3233	0.0408

Supplementary Table 10. Comparison of linkage disequilibrium (LD) in the region surrounding *SiLes1* vs. *SvLes1*. Intervals as in Table S9.

				Setaria viridis			***Setaria italica***		
**Description**	**Start**	**End**	**Length (bp)**	**Mean**	**% of comp. (>=0.5)**	**% of comp. (>=0.7**	**Mean**	**% of comp. (>=0.5)**	**% of comp. (>=0.7**
Gene	6847970	6850236	2266	0.23	22.07	20.69	0.80	100.00	100.00
10kb total	6844103	6854103	10000	0.23	19.37	14.60	0.96	100.00	100.00
20kb total	6839103	6859103	20000	0.26	24.78	15.28	0.97	100.00	100.00
50kb total	6824103	6874103	50000	0.29	27.17	20.68	0.30	25.91	25.91
10kb either side	6837970	6860236	22266	0.27	25.63	17.36	0.98	100.00	100.00
20kb either side	6827970	6870236	42266	0.28	25.63	19.51	0.30	25.91	25.91
50kb either side	6797970	6900236	102266	0.27	22.68	16.64	0.46	42.35	42.13

Supplementary Table 11. Genomic libraries included in the *Setaria viridis* genome assembly and their respective assembled sequence coverage levels in the final release.

*Average read length of PACBIO reads.

**Library**	**Sequencing Platform**	**Assembly Release**	**Average Read/Insert Size**	**Read Number**	**Assembled Sequence Coverage (X)**
H0022	Illumina	2.0	800	425,635,116	240
	PACBIO	2.0	10,587*	4,768, 857	118.18

Supplementary Table 12. PACBIO library statistics for the libraries included in the *Setaria viridis* genome assembly and their respective assembled sequence coverage levels.

Cutoff	Number of Reads	Basepairs	Average Read Length	Coverage
0	4,768,857	59,091,859,660	10,587	118.18x
1,000	4,427,654	58,922,852,295	11,597	117.85x
2,000	4,128,899	58,477,661,257	12,522	116.96x
3,000	3,875,244	57,847,410,625	13,346	115.69x
4,000	3,653,717	57,073,702,495	14,086	114.15x
5,000	3,442,812	56,125,591,515	14,820	112.25x
6,000	3,238,591	55,003,123,843	15,551	110.01x
7,000	3,041,062	53,719,962,620	16,270	107.44x
8,000	2,850,014	52,287,501,523	16,979	104.58x
9,000	2,665,092	50,716,192,061	17,681	101.43x
10,000	2,486,248	49,017,650,265	18,373	98.04x
11,000	2,314,198	47,211,798,377	19,049	94.42x
12,000	2,148,024	45,301,509,765	19,727	90.60x
13,000	1,990,291	43,330,677,398	20,385	86.66x
14,000	1,839,724	41,298,472,587	21,043	82.60x
15,000	1,696,209	39,218,281,557	21,692	78.44x
16,000	1,557,328	37,065,930,006	22,354	74.13x
17,000	1,422,360	34,839,361,404	23,034	69.68x
18,000	1,291,347	32,547,099,163	23,740	65.09x
19,000	1,163,251	30,177,734,218	24,475	60.36x

Supplementary Table 13. Summary statistics of the initial output of the QUIVER polished MECAT assembly. The table shows total contigs and total assembled basepairs for each set of scaffolds greater than the size listed in the left hand column.

Minimum Scaffold Length	Number of Scaffolds	Number of Contigs	Scaffold Size	Basepairs	% Non-gap Basepairs
5 Mb	25	25	347,994,520	347,994,520	100.00%
2.5 Mb	33	33	378,539,963	378,539,963	100.00%
1 Mb	40	40	389,332,751	389,332,751	100.00%
500 Kb	44	44	392,513,339	392,513,339	100.00%
250 Kb	49	49	394,501,069	394,501,069	100.00%
100 Kb	53	53	395,090,738	395,090,738	100.00%
50 Kb	77	77	396,626,132	396,626,132	100.00%
25 Kb	107	107	397,813,004	397,813,004	100.00%
10 Kb	110	110	397,864,082	397,864,082	100.00%
5 Kb	110	110	397,864,082	397,864,082	100.00%
2.5 Kb	110	110	397,864,082	397,864,082	100.00%
1 Kb	110	110	397,864,082	397,864,082	100.00%
0 bp	110	110	397,864,082	397,864,082	100.00%

Supplementary Table 14. Final summary assembly statistics for chromosome scale assembly. Scaffold sequence total is all bases in the release plus gaps. Chromosome sequence is all bases in the chromosomes not including gaps. Total bases is all bases in the release excluding gaps.

**Scaffold total**	14
**Contig total**	75
**Scaffold sequence total**	395.7 Mb
**Chromosome Sequence**	394.9 Mb
**Total bases**	395.1 Mb
**Scaffold N/L50**	4 / 46.7 Mb
**Contig N/L50**	11 / 11.2 Mb
