# Supplementary material for: A genome resource for green millet Setaria viridis enables discovery of agronomically valuable loci
Source: Nat Biotechnol. 2020 Oct 5;38(10):1203–10. doi: 10.1038/s41587-020-0681-2 (PMC7536120; doi:10.1038/s41587-020-0681-2)
Supplement: Supplementary file 2 — Reporting Summary [file 41587_2020_681_MOESM2_ESM.pdf]

nature research

Corresponding author(s): Elizabeth A. Kellogg

Last updated by author(s): Aug 12, 2020

Reporting Summary

Nature Research wishes to improve the reproducibility of the work that we publish. This form provides structure for consistency and transparency in reporting. For further information on Nature Research policies, see our Editorial Policies and the Editorial Policy Checklist.

Statistics

For all statistical analyses, confirm that the following items are present in the figure legend, table legend, main text, or Methods section.

n/a	Confirmed	
☐	☒	The exact sample size (n) for each experimental group/condition, given as a discrete number and unit of measurement
☐	☒	A statement on whether measurements were taken from distinct samples or whether the same sample was measured repeatedly
☐	☒	The statistical test(s) used AND whether they are one- or two-sided<br>*Only common tests should be described solely by name; describe more complex techniques in the Methods section.*
☒	☐	A description of all covariates tested
☐	☒	A description of any assumptions or corrections, such as tests of normality and adjustment for multiple comparisons
☐	☒	A full description of the statistical parameters including central tendency (e.g. means) or other basic estimates (e.g. regression coefficient) AND variation (e.g. standard deviation) or associated estimates of uncertainty (e.g. confidence intervals)
☐	☒	For null hypothesis testing, the test statistic (e.g. F, t, r) with confidence intervals, effect sizes, degrees of freedom and P value noted<br>*Give P values as exact values whenever suitable.*
☒	☐	For Bayesian analysis, information on the choice of priors and Markov chain Monte Carlo settings
☒	☐	For hierarchical and complex designs, identification of the appropriate level for tests and full reporting of outcomes
☒	☐	Estimates of effect sizes (e.g. Cohen's d, Pearson's r), indicating how they were calculated

Our web collection on statistics for biologists contains articles on many of the points above.

Software and code

Policy information about availability of computer code

Data collection	Described in Methods. All open source or commercially available and previously published.
Data analysis	Described in Methods. All open source or commercially available and previously published.

For manuscripts utilizing custom algorithms or software that are central to the research but not yet described in published literature, software must be made available to editors and reviewers. We strongly encourage code deposition in a community repository (e.g. GitHub). See the Nature Research guidelines for submitting code & software for further information.

Data

Policy information about availability of data

All manuscripts must include a data availability statement. This statement should provide the following information, where applicable:

- Accession codes, unique identifiers, or web links for publicly available datasets
- A list of figures that have associated raw data
- A description of any restrictions on data availability

Data availability: Sequences are available in the SRA at NCBI, BioProject number PRJNA527194. Seed stocks for diversity lines are available by contacting co-authors I. Baxter or E. A. Kellogg.

Field-specific reporting

Please select the one below that is the best fit for your research. If you are not sure, read the appropriate sections before making your selection.

☐ Life sciences ☐ Behavioural & social sciences ☒ Ecological, evolutionary & environmental sciences

For a reference copy of the document with all sections, see nature.com/documents/nr-reporting-summary-flat.pdf

Ecological, evolutionary & environmental sciences study design

All studies must disclose on these points even when the disclosure is negative.

Study description	Production of a high-quality reference genome; population genetics and genome-wide association study of natural diversity; coning and validation of an agronomically important gene for shattering; identification of other loci of possible agronomic importance.
Research sample	Wild accessions of Setaria viridis were collected throughout the US over a period of several years. One such accession ME034V has been used frequently for transformation experiments so we included that one. The reference line is Al0.I.
Sampling strategy	Sample size was determined by the amount of time and money we could devote to collecting, which ultimately led to 605 accessions. The sample size for GWAS was chosen based on the numbers of sequenced individuals that we had available at the time (c. 200), and was sufficient to identify one large QTL.
Data collection	See Methods. Data include sequence data plus phenotypic data on transgenic plants.
Timing and spatial scale	Spatial scale covered North America during the normal growing season of S. viridis, which is late July to October. Other experiments were done in the greenhouse.
Data exclusions	Sequences of 7 accessions were excluded because of poor quality.
Reproducibility	All experiments were replicated. Numbers of replicates vary, but generally 2 to 4 times.
Randomization	Randomization is not relevant for the investigations reported here.
Blinding	Specimens were assigned to groups for tensile strength measurements and wind tunnel experiments based on their genotype at the Lesl locus. Because this phenotype is hard to detect by eye, we did not think that blinding was necessary.

Did the study involve field work? ☒ Yes ☐ No

Field work, collection and transport

Field conditions	Plant were collected throughout the growing season in North America. Seeds from the field grown plants were returned to the Danforth Plant Science Center and planted. Leaf material was either harvested from these first generation plants, or the accession was propagated by single-seed descent and leaf material harvested from later generations.
Location	Latitude and longitude was recorded for all field collected plants. Specific localities are shown in the maps in Figure 2. Experimental work was done in St. Louis, MO.
Access & import/export	Setaria viridis is a weed and grows along highways. We did not collect in parks or other protected areas .. The Canadian accessions were imported to the US with appropriate permission. Accessions from Eurasia were obtained from collaborators or seed banks.
Disturbance	Although plants were collected roots and all, they are shallow rooted and grow in naturally disturbed sites along roads and highways. They occur in large populations, and we sampled only 3-5 plants per population.

Reporting for specific materials, systems and methods

We require information from authors about some types of materials, experimental systems and methods used in many studies. Here, indicate whether each material, system or method listed is relevant to your study. If you are not sure if a list item applies to your research, read the appropriate section before selecting a response.

Materials & experimental systems

n/a	Involved in the study
☒	☐ Antibodies
☒	☐ Eukaryotic cell lines
☒	☐ Palaeontology and archaeology
☒	☐ Animals and other organisms
☒	☐ Human research participants
☒	☐ Clinical data
☒	☐ Dual use research of concern

Methods

n/a	Involved in the study
☒	☐ ChIP-seq
☒	☐ Flow cytometry
☒	☐ MRI-based neuroimaging
